# Asparaginase treatment side-effects may be due to genes with homopolymeric Asn codons (Review-Hypothesis)

**DOI:** 10.3892/ijmm.2015.2285

**Published:** 2015-07-15

**Authors:** JULIAN BANERJI

**Affiliations:** Center for Computational and Integrative Biology, MGH, Simches Research Center, Boston, MA 02114, USA

**Keywords:** asparaginase, diabetes, lipodystrophy, leukemia, lymphoma, immune response, pancreatitis, cystic fibrosis, insulin regulatory substrate-2, *Salmonella*

## Abstract

The present treatment of childhood T-cell leukemias involves the systemic administration of prokary-otic L-asparaginase (ASNase), which depletes plasma Asparagine (Asn) and inhibits protein synthesis. The mechanism of therapeutic action of ASNase is poorly understood, as are the etiologies of the side-effects incurred by treatment. Protein expression from genes bearing Asn homopolymeric coding regions (N-hCR) may be particularly susceptible to Asn level fluctuation. In mammals, N-hCR are rare, short and conserved. In humans, misfunctions of genes encoding N-hCR are associated with a cluster of disorders that mimic ASNase therapy side-effects which include impaired glycemic control, dislipidemia, pancreatitis, compromised vascular integrity, and neurological dysfunction. This paper proposes that dysregulation of Asn homeostasis, potentially even by ASNase produced by the microbiome, may contribute to several clinically important syndromes by altering expression of N-hCR bearing genes. By altering amino acid abundance and modulating ribosome translocation rates at codon repeats, the microbiomic environment may contribute to genome decoding and to shaping the proteome. We suggest that impaired translation at poly Asn codons elevates diabetes risk and severity.

## 1. Foundation of the hypothesis

### Core hypothesis: translocation rates, poly Asparagine (Asn); insulin-receptor-substrate 2 (IRS2) and diabetes; hypothesis tests, poly glutamine (Gln) HTT and ataxias

**Figure 1:**
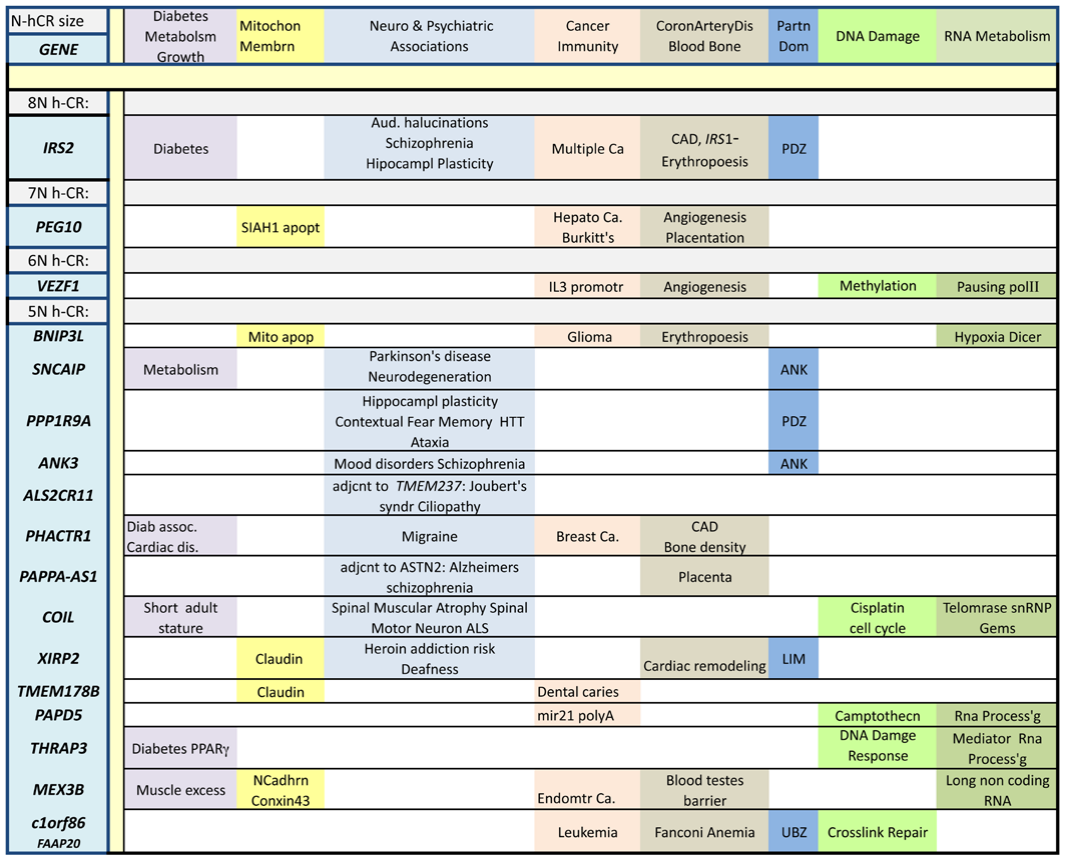
Asn homopolymeric coding regions (N-hCR)-bearing-genes from 8N-hCR to 5N-hCR. The 17 human genes with N-hCR of length greater than five. Human genes are grouped by N-hCR length. Rows list genes, labelled on the left and grouped by N-hCR length in descending order from insulin-receptor-substrate 2 (*IRS2*) with 8N-hCR. Columns of colored panels suggest (manually annotated) functional categories: purple, fiabetes and metabolism; yellow, membrane and mitochondria; blue, neuro; pink, cancer and immunity; grey, cardiovascular, blood and bone; green, DNA/RNA. Karlin *et al* (1) have speculated that N-hCR shorter than five in length would arise by chance. However, Kriel and Kriel (2) demonstrates that the statistical difference between mammals and nonmammals continues to hold at least down to 3N-hCR. The cutoff threshold of significance would then reduce to 2N-hCR, and to the definition of a transcription unit, *cf*. *VEZF1*, which has multiple cDNAs defining infrequently used exons. N.B. Adjacent, potentially cojoined (380) genes are used to categorize *PAPPA-AS1* and *ALS2CR11*. Like the *PAPPA* locus, the *MEPC2* locus also has an N-hCR bearing antisense transcript, with a 7N-hCR (AF361491); The metabolic disease and retinal development associated gene *SIX3* has an antisense N-hCR bearing transcript in human *SIX3-AS1* (NR_1037686.1) and mouse *SIX3-OS1* (NR_038083.1). SNP rs16882396 marks the association of periodontal disease with *TMEM178B*. The 49 genes with 4N-hCR are: *ACACA, ACACB, AGBL2, BAI2, BMPR2, C2orf61, CD9, CFTR, CHRM2, CNOT10, EOMES, EPPIN, EPPIN-WFDC6, EVI2A, FAM193A, FRS3, GTF2I, IL9R, KIAA1841, KIF3C, KLF17, LEMD3, LRP6, MAML2, MYRF, NCOA1, PARP3, PEAK1, PPP1R13B, RNF103, SH3D19, SI, SLIT1, SLIT2, SLIT3, SNAP91, TAB2, TAB3, TAX1BP1, TEC, TMEM57, TOX3, TRPM6, TRPM7, TTC8, TTLL5, UBE4A, ZXDA, ZXDB* Unorthodox human proteins deserving closer attention are from unusual cDNAs: *Map3K2*_4N-hCR_ AAH65755.1; *TCRα*_5N-hCR_ AIE11180.1; *Vκ*_5N-hCR_ AAO11865; and *Vλ*_4N-hCR_ AAD29331.1. The germline V regions of immunoglobulin (Ig) λ as well as T cell receptor AlphaJ regions are represented in Table I as 3N-hCR. However, there are rearranged cDNAs encoding for up to 5N-hCR in some hypervariable regions (HVR) that do not appear in the germline N-hCR (used for assigning length of N-hCR when classifying these genes). It is unclear what benefits, if any, could accrue to an Ig synthesized and, potentially, folded at a rate regulated by Asn levels at N-hCR. An arbitrary list of genes that may respond to fluctuations in other amino acids include *CNDP1, CYP21A2, SELT, SELM* (L-hCR); *CACNA1D* (M-hCR); *HSD11B1* (Y-hCR); *NR4A3* (H-hCR); *TAF9, URI1, ASPN, EFTUD2, GLTSCR1L, THBS4* (D-hCR); *HRC* (D-, E-, H-hCR); *ATAD2* (S-, D-hCR); *EIF5B* (K-, D-, E-hCR); *KCNMA1, MAP3K1, CXXC4, WDR26, TNRC18, SRRM2* (S-, T-, G-hCR); *CACNA1A* (H, N, Q-hCR); *POU4F2* (M-, G-, H-, S-hCR); *POU3F2* (G-, H-, Q-hCR); *SKIDA1* (H-, E-, A-hCR); *USP34* (H-, N-hCR); *ATXN1, ATXN2, ATXN3, ATXN7, AR, KMT2D, KMT2C, MAMC2, MAML3, FOXP2, ARID1A, ARID1B, ARID3B MED12, MED15, NCOA3, NCOA6, IRF2BPL, VEZF1, ABCF1* and *HTT* (Q-hCR). The hCR appear in proteins from the NCBI homologene (381) database.

Despite similar Asn codon usage, ~4%/gene, from plants to humans (1), mammals are distinguished by a paucity of genes with a long Asn homopolymeric coding region (N-hCR) (2). The 17 human genes with the longest N-hCR (ranging from five to eight consecutive Asn codons) are listed in Fig. 1; Table I lists genes with N-hCR greater than three. *IRS2*, encoding an insulin signal transducer, is the gene at the top of the list in Fig. 1 and multiple disorders of energy homeostasis and the urea cycle are associated with genes in Table I. The central hypothesis of this paper is that manifestations of these disorders may partly be attributable to reduced plasma Asn concentrations, which in turn may disproportionately affect the production of proteins containing N-hCR. More broadly, we propose a model in which protein expression may be affected at amino acid homopolymeric coding regions (hCR) in general because translation elongation rates at hCR could reflect variation in the levels of the corresponding amino acids. This model may contribute to explaining an association, initially noted with poly Gln codon runs, between hCR and some human diseases (1,3).

Asparaginase (ASNase) is a component of highly effective chemotherapeutic regimens used to treat pediatric acute lymphoblastic leukemia (ALL) (4,5) and some lymphomas (6–8). ASNase treatment has been estimated to have contributed to the sparing of the lives of upwards of 60,000 children in the US in the decades following its discovery (9) and rapid introduction to the clinic (10). However, ASNase treatment is not without hazard; it can produce a myriad of side-effects that include hyperglycemia, dislipidemia, pancreatitis, vascular accidents and adverse neurological outcomes. The physiological mode of action of ASNase is unclear. The enzyme deaminates Asn and Gln with production of altered amino acid ratios and ammonia (11–15). ASNase inhibits synthesis of proteins *in vitro* (16) and *in vivo* (17,18) by a mechanism consistent with reduced ribosomal translocation at Asn codons. In humans, ASNase treatment protocols cause depletion of plasma Asn and modest reductions of plasma Gln levels accompanied by mild transient hyperglycemia and occasional ketoacidosis (11,19,20). In mice, administration of ASNase causes Asn depletion in plasma and some tissues, e.g., skeletal muscle (21,22), indicating, importantly, that intracellular Asn can also be depleted. Moreover, in mice, impaired glucose tolerance following ASNase treatment can be improved by amino acid supplements which serve to moderate amino acid ratio imbalances (23) and Asn administered directly to mice reverses adverse events initiated by ASNase (24). In rabbits, ASNase induces dose-dependent glycemic dysregulation extending from transient mild glycosuria to hyperglycemia and diabetes (25,26). Prednisolone has been shown to potentiate the action of ASNase: both drugs can cause hyperglycemia when used alone; but predisolone synergizes with ASNase to cause significant hyperglycemia (500–700 mg/dl) when both drugs are administered in combination at doses that are insufficient to produce an effect above baseline (~100 mg/dl) when either drug is administered alone (27).

Complementing these clinical and experimental observations, metabolomic data from the Framingham Heart study and from diabetic patients in a Shanghai study have shown that plasma Asn concentration is negatively correlated with fasting insulin concentration (28), and that the degree of negative correlation is the highest for Asn by comparison with the 20 amino acids that are commonly incorporated into proteins by ribosomal synthesis. By contrast, γ-amino butyric acid (GABA) levels are 10-fold more negatively correlated with fasting insulin levels. In the Framingham data, the maximal negative correlation observed between Asn concentration and fasting insulin also extends to additional diabetes metrics such as body mass index (BMI), waist circumference (WC), homeostatic model assessment (HOMA), and triglyceride levels. In a third study, of a different cohort, Asn was the amino acid most negatively correlated with adiponectin, HOMA and leptin levels (29). Because therapeutic Asn depletion induces glycemic dysregulation, low Asn levels may not merely be correlatively associated with poor glycemic control, but may be causative or provocative. This raises the question of the potential mechanisms by which Asn depletion in plasma or tissues could adversely impact glucose homeostasis.

The possibility that N-hCR can be implicated in the etiologies of some diabetic syndromes is supported by the enrichment of genes governing metabolic balance among the list of those containing N-hCR. Approximately one-fifth of the genes bearing N-hCR in Table I are associated with metabolic disorders, obesity, diabetes, urea cycle or pancreatic islet β-cell regulation. Among these, *IRS2* is of particular note. *IRS2* encodes insulin receptor substrate-2, a labile (30,31) intracellular signal transducer that is a substrate for a number of membrane spanning receptor tyrosine kinases specific for extracellular cytokines that include insulin, insulin-like-growth-factor-1, erythropoietin, thrombopoetin, growth hormone, leukemia inhibitory factor, interleukin-4 (IL-4) and interferon-γ (32–37). Sequence polymorphisms in the human *IRS2* locus have been associated with obesity (38), type 2-diabetes-mellitus (T2DM) (39,40) or its complications (41,42), aspects of schizophrenia (43) and IgE immune responses (44). In transgenic mice, *IRS2* deletion causes compromised maintenance of β-cell mass and produces a diabetic state similar to T2DM (45,46). Reduced levels of IRS2 in humans have been proposed to lead to desensitized insulin/cytokine signalling and thus to hyperglycemia/muted immune responses, with prolonged IRS2 deficits exacerbating islet cell mass reduction leading to T2DM (47–50). Alterations in IRS2 expression have been associated with altered lipid metabolism in obese subjects (51) and have been correlated with development of insulin nonresponsiveness in obese boys (52). IRS2 has eight consecutive Asn-codons located 19 codons after the initiator AUG codon. Depletion of the levels of the cognate Asn aminoacyl-tRNA may result in compromised elongation in the homopolymeric Asn coding region that may be especially deleterious to the synthesis of IRS2 due to the location of the N-hCR.

Codon usage and ribosome translocation rates affect protein expression in bacterial (53–57), viral (58,59) and human genes (60,61). Ribosomal footprinting studies have suggested that the stability of translation initiation complexes increases when nascent chains emerge from the exit tunnel or folding vestibule to engage chaperones (62). Ribosomal stalling may potentially lead to translation termination when the elongation rate is diminished in the 'translation-initiation-ramp' or instability region (63–65). The concept of the ramp, which may not apply to all mammalian genes, remains controversial (66) and though potentially contributory, it is not essential to the overall thesis proposed here. In general, a severely diminished elongation rate may lead to premature termination; for example in prokaryotes, ribosomal stalling induces a translational termination mechanism through tmRNA (67, *Cf*. 68). In the abstract, reduced rates of translation anywhere along an mRNA would result directly in a reduced overall rate of target protein synthesis and, depending on protein halflife, result indirectly in decreased steady state levels of such proteins. High rates of translation may even increase the halflife of an mRNA (69).

Of the genes that have been identified with N-hCR of length 3 or greater, approximately one third can be associated with cancer and immune response, one quarter with neurode-generation (20% with metabolic disorders, above), and eight percent with vasculature and hematopoesis. Of the remaing ~14%, many can be classified as involved with chromatin modification, DNA maintainance and repair, RNA transcription and processing or protein synthesis and turnover, some have Leucine rich repeats that can serve as pattern recognition elements. Some genes fall into multiple categories, e.g. *IRS2* is associated not only with diabetes and receptor mediated signal transduction for specific extracellular cytokines, but also with epilepsy (70), aspects of schizophrenia (43), Alzheimer's disease (71–73), retinal degeneration (74), hippocampal synaptic plasticity (75), long term potentiation of hippocampal synaptic transmission (76), ataxia (77), cardiac failure (78), kidney development (79), renal disease (80), breast cancer (81,82), rhabdomyosarcoma (83) and, in conjunction with *JAK2*_3N-hCR_, hematopoesis (84,85). A limited study of an N-hCR length polymorphism in *IRS2* shows no association with diabetes (86).

For the purpose of establishing the consequences of N-hCR for translational sensitivity to Asn concentration, other genes with N-hCR could be tested, including conserved genes with nonhuman N-hCR lengths that also differ from humans in some other parameter (such as inflammatory response profiles) (87). For example an exceptional mammalian gene, with an N-hCR longer than the 8N-hCR of IRS2, is a bat paralog of the IL8-receptor, *CXCR2*, (EPQ18419), which has a 60N-hCR. Other genes of interest from mouse, that differ from human in N-hCR length, include *MDR1* and *CFTR* (a *Salmonella* receptor), and *TNFRSF16/BEX3A/NGFRAP1* (implicated in diabetes) (88) as well as the redox regulators: *GCLC* (89) and *TXNIP* (90) (the former encodes the first, rate limiting, enzyme in the glutathione synthesis pathway and has been associated with cardiovascular events) (91); the latter encodes a conserved thioredoxin binding protein that has an 8N-hCR in mice, vs. a 3N-hCR in nonrodent mammals. All of these *TXNIP* N-hCR are invariantly located and they begin at codon 386, end 3 codons before the stop codon. This is discussed further, below, along with the contribution of TXNIP to host response to *P. aeruginosa* bacteremia by recruitment of neutrophils in mice (92). TXNIP also affects pancreatic β-cell biology (93), diabetic retinopathy (94), and glucose metabolism (indirectly regulated by mTOR) (95). Finally, a gene with the third longest N-hCR in the mosquito genome (XM_316513) is translationally regulated (perhaps at its N-hCR) in insect midgut in response to plasmodium infected blood meals (96). The gene is homologous to human *FAF1/TNFRSF6* which is associated with diabetes (97) and Parkinson's disease (PD) (98).

Human genes with hCR have been linked to complex diseases (1). Genes that may respond to fluctuations in amino acids other than Asn (99–106), include *CNDP1* (107,108) (L-hCR), *MEPC2e1* (109) (A,G,H-hCR), and *HTT* (Q-hCR) (110). The gene list could also extend to *DMPK/SIX5* (111,112), *GCLC* (89), *FMR1* (113) and *C9orf72* (114–116) if unorthodox, repeat-associated-non-ATG (RAN), translation of upstream codon repeats (117–120), or alternate transcript variants (121) are included.

The *HTT* locus mediates the deleterious effects of Huntington's ataxia, and is one of the early examples of a gene containing an hCR associated with a disease (122). It has a Q-hCR whose length can vary inversely with the age of onset and severity of the ataxia. The 23Q-hCR of HTT is situated in its ramp region, with a 16 codon interval between the hCR and the initiator AUG. Although much of the effort to understand Huntington's disease has focused on aggregation of products of the HTT locus (123,124), the etiology of truncated translation products resulting from ribosomal stalling in the Q-hCR has received much less attention. Exon truncation fragments may arise if HTT is expressed in an environment of limiting Gln (22,125) and the resulting increase in neuronal cell death (126), could accelerate the onset and clinical course of Huntington's disease (127,128).

## 2. ASNase produced by the biome. The potential for N-hCR-bearing-genes to cause side-effects

### ASNase production by Salmonella, pancreatitis, immunosupression

Genetic studies suggest an environmental component for the etiology of diabetes (129) and the gut microbiome has been proposed to regulate human physiology, e.g. bone mass (130). An individual's microbiome may also produce enzymes that alter host Asn levels. Persistent salmonellosis in mice causes pancreatitis (131,132) which is a side-effect of therapeutic ASNase treatment (133,134). In addition, *Salmonella* mediates its own virulence (135) via a cytostatic ASNase (16) and inhibits mouse T cell responses in a manner reversible by administration of Asn (24,136); this *Salmonella* mediated immune inhibition may reflect the immunosuppression noted in ASNase-treated rabbits (137) and rodents (138,139).

### Elongation: pancreatitis, cystic fibrosis, dislipidemia, clotting, complement and neurodysfunction; Notch, WNT and hedgehog

Allelic variation in loci encoding-N-hCR-bearing-genes, such as *KCNA3*, *CFTR*, *SLC26A9*, *SCARB1*, *IRS2*, *F5*, *FGB* and *SHANK1*, have been associated with diabetes, pancreatitis, lipidystrophy, vascular disorders and neurological changes (140–144). *KCNA3*3N-hCR encodes a potassium channel that has allelic variants associated with altered risk for ALL (145) in a certain (germ line *RUNX* rearranged) subset of children and its mouse homolog regulates energy homeostasis and body weight (146). KCNA3 is thought to have its structure and function affected during its synthesis by residence time of certain of its elongating domains in the ribosomal vestibule (147–149) (*cf*. *KCNH4*_3N-hCR_ and *KCNH*8_3N-hCR_). Pancreatitis and diabetes are associated, respectively, with *CFTR*_4N-hCR_ and *SLC26A9*_3N-hCR_, the products of which physically and functionally interact. CFTR is an ion channel, closely related, by membership in the superfamily of ATP-binding cassette proteins, to the multidrug resistance transporter (MDR1) (150–153). Some *MDR1* alleles contain a polymorphic synonymous codon substitution at Gly412 (C1236T), very similar in location to Asn416 in the N-hCR of *CFTR*. Such polymorphisms in *MDR1* have been proposed (154) to affect its rate of translation elongation resulting in alterations in the conformation of MDR1 with concomitant functional changes in the profile of anticancer drugs that MDR1 transports (60). The N-hCR of *CFTR*, located in the regulatory insert (RI) between the membrane spanning domain (MSD) and the nucleotide binding domain (NBD) could, by analogy to the key *MDR1* Gly412 substitution, alter translation rate at its Asn 415 to 418 region, under conditions of low Asn, to result in generation of CFTR protein folding variants (155) with altered function that may affect bicarbonate exchange (in co-assemblies with *SLC26A9*_3N-hCR_) (156–158), *Salmonella* susceptibility (159), and timing of cystic fibrosis (CF) disease onset (160).

A similar location of N-hCR, between MSDs and NBDs, is found in two genes that encode important ATP-regulated magnesium channels: *TRPM6*_4NhCR_ and *TRPM7*_3N-hCR,4NhCR_. Allelic variation of the former has been associated with elevated risk of diabetes, osteoporosis, asthma, and heart and vascular diseases (161), whereas allelic variation of the latter has been associated with sudden cardiac death, QT interval prolongation and atrial fibrillation in individuals with African ancestry (162), and ALS and PD in Guam (163). TRPM6 can form heterodi-mers with, and regulate function of, TRPM7; the latter is a channel regulated enzyme that can be cleaved to modify histones (164,165). TRPM7 affects vascularization (166), and has been implicated in ovarian, breast, pancreatic and prostate cancer as well as in the metastasis of nasopharyngeal carcinoma (167). The NBDs of these ion channels, as well as the STAS domain of *SLC26A9*_3N-hCR_ (151) (which is thought to assemble and interact with the Regulatory domain in the NBD of CFTR), all have poly Asn regions separating them from portions of their hydrophobic MSDs, suggesting that translocation rate at the N-hCR, perhaps due to variation in Asn levels, may serve to modulate the chronology of the synthesis and assembly of the hydrophobic intracellular domains of these molecules.

Dislipidemia could be caused by altered translation of *SCARB1*_3N-hCR_. A list of fifteen candidate genes in which synonymous codon substitutions may be of functional consequence, perhaps due to altered translation rate affecting protein synthesis, includes not only *MDR1* (Gly412 and Ile1145) but also *CFTR* (Ile507 and ΔF508) (160) and *SCARB1* (Ala350) (168). Rs5888, a synonymous substitution in *SCARB1* of codon Ala350, adjacent to Asn349, is associated with increased risk of coronary artery disease (CAD) and ischemic stroke (169–171). Translation rates of *CFTR* and *SCARB1* may be regulated not only at the synonymous codon substitutions above, but also, in response to Asn concentration changes, at their N-hCR. SCARB1 is a high density lippoprotein (HDL) receptor that participates in lipid metabolism and flux of cholesterylesters (172) into e.g. HDL particles that contribute to cell signalling (173) and thus it could mediate the dislipidemia that accompanies the therapeutic administration of ASNase (174). *SCARB1* affects suseptibility to myocardial infarction (175) and renal cell carcinoma (176,177) activity of lippoprotein associated phospholipase A2 (Lp-PLA2) (178), and causes an anti-inflammatory effect in macrophage (179); it indirectly affects atherosclerosis (180), mitigates stress (181), and affects fertility (182) and macular degeneration (183). By influencing gut absorption of vitamins, it can affect vascular integrity and diabetes suseptibility (184–188). A similar synonymous codon substitution at Cys816 of IRS2, (rs4773092), is associated with an auditory component of schizophrenia (43); this supports the notion, with the usual caveats regarding RNA stability, that *IRS2* may also be translationally regulated, for example at its N-hCR.

ASNase treatment produces side-effects that include vascular dysfunction. Factor V and fibrinogen are two of several coagulation and complement factors encoded by N-hCR-bearing-genes. Polymorphic alleles of *F5*_3N-hCR104t_ (encoding coagulation Factor V) have been linked to coronary artery disease (189), hippocampal degeneration (190) and thrombotic events in ASNase treated children (144,191). ASNase specifically reduces the synthesis rate of fibrinogen (18), see below, a subunit of which is encoded by *FGB*. Thus inhibition by ASNase of the synthesis of at least two N-hCR-bearing-genes, *F5* and *FGB*, could potentially account for the vascular side-effects of ASNase administration. *FGB*_3N-hCR_, *GP1BA*_3N-hCR_, encoding the platelet membrane receptor (for von Willebrand's factor) associated with ischemic stroke (192), and *CD9*_4N-hCR_, a gene involved in platelet formation (193), are candidate N-hCR bearing genes that could be examined for their genetic association with adverse vascular events attending ASNase treatment (as has been reported for F5, above). Coagulation proteins have long been considered potential risk factors of ASNase therapy (194). The steady state half-life of autologous iodinated fibrinogen is not affected by ASNase treatment and hence the observed reduction in steady state plasma fibrinogen concentration that produces the hypofibrinogenemia (195) observed after ASNase treatment is likely due to inhibition of fibrinogen synthesis (18). There are concordant studies in rabbits (196) and humans (197) regarding the rate of catabolism and synthesis of fibrinogen in response to ASNase, as well as studies on the proteomics of FGB and C3 in diabetics (198,199). N-hCR-bearing-genes encoding complement proteins may also contribute to other disorders such as retinal degeneration through effects on *C3*_3N-hCR_ (200) to multiple sclerosis through effects on *C7*_3N-hCR_ (201) and to uptake of pathogens such as glycosylated viruses or bacteria by any of multiple members of the lectin and alternate complement pathway on Table I such as *CLEC6A* (202), *CLEC10A* (203) *CLEC13B/LY75*, *MASP1* and *C1QB*.

Mitigating the effects of low plasma Asn, by altering the composition of intestinal microbiota (204) or by using amino acid supplements (23), may slow disease onset or progression in those at risk of diabetes or its complications. Dietary Asn supplementation may particularly benefit CFTR-null homozygotes or compound heterozygotes, who frequently present with diabetes at later stages of their disease (205). One of the N-hCR-bearing-genes in Fig. 1, *PHACTR1*_5N-hCR_ has been linked to coronary artery disease (CAD) in diabetics (206). Diabetes and CAD are frequent comorbidities, as are diabetes and Alzheimer's disease (72) perhaps due to a shared etiology originating in low plasma Asn concentration. There are two N-hCR-bearing-genes from Fig. 1 that are linked to PD and mood disorders: *SNCAIP*_5N-hCR_ and *ANK3*_5N-hCR_. PD and diabetes are comorbidities, and abnormal glucose regulation has been reported in >50% of PD patients (207) perhaps due to altered Asn homeostasis; correspondingly, bipolar disorder treatment outcomes differ for patients with diabetes as compared to normal controls (208). PD and ALS often occur with dementia (209,210); a shared etiology may be responsible, due to altered levels of Asn, perhaps even through complement genes such as *C1QB*_3N-hCR_ (211), or the balance between *C1QL2*_3N-hCR_, *C1QL3*_3N-hCR_ (212) and *BAI2*_3N-hCR_ and their non N-hCR bearing paralogs: *C1QL1* and *BAI3* (213).

Multiple genes encoding N-hCR have been linked to neuropsychiatric disorders, PD, aspects of schizophrenia, Alzheimer's disease, mood disorders [*CDH9* (214), *GTF2I* (215) and *ALDH6A1*], neurological dysfunction (*CDKL5* and *TMEM106B*) (216,217), breast-cancer [*BRCA2*, *CEACAM5/CEA* (218), *CYP19A1*/Aromatase (219), *IRS2*, *CLEC10A* (220), *LRP6* and *TBC1D5* (221)], spinal degeneration (*COIL*, *FBXO38*, *ITGAV*, *ASIC2*, *KIAA1217* and *CHAD*), age of onset of amyotrophic lateral sclerosis (ALS) (*TTLL4* and *LAMA3*) (222), dementia in ALS (*TMEM106B*) (223) retinal dystrophy (*TTLL5*) (224), large artery stoke (*TTLL5* and *PHACTR1*) (225) decreased bone density in tamoxifen treated women (*LRP4* and *NCOA1*) (226), ovarian cancer (*TBC1D3* and *TBC1D3F*) (227) T cell anergy (*GRAIL/RNF128/isf2*) (228–230), asthma, autoimmune diseases, innate immunity (231–233) and the link between innate and adaptive immunity (*FCGR2-A, -B, -C*) (234) suggesting a common etiology of altered Asn homeostasis may need to be considered for some of these conditions.

LRP5, LRP6 and APC are encoded by N-hCR-bearing-genes involved in the Wnt pathway. Rotterlin, which is reported to accelerate the turnover rate of LRP6 (235) (a Wnt signalling co-receptor) (236), could be co-administered with ASNase because it may potentially synergize with ASNase to focus the effect of ASNase on LRP6 mediated Wnt signalling (237). We hypothesize that by preferentially lowering the steady state level of LRP6, the combination of drugs could regulate (238) bone mass, cancer, cardiovascular health, vision, Alzheimer's and multiple other diseases of aging. Notch and hedgehog signalling are also affected by N-hCR bearing-genes such as *DZIP1*, *MAML2*, *BOC* and *CDON*, and may present attractive targets for drug discovery via small molecules that accelerate turnover of specific proteins encoded by N-hCR bearing-genes, synergistically magnifying the impact of ASNase by altering the replacement rate and perhaps by establishing lowered steady state levels of the targeted protein. There is already a precedent for synergism of prednisolone with ASNase, which occurs by an as yet unknown mechanism. The halflife of WNT signalling complexes and the contribution of DSV to turnover of WNT coreceptors FZD and LRP6 has recently been characterized (239).

The psychiatric disorders associated with ASNase treatment of adults (240) have been ascribed to ammonia toxicity and cerebrovascular-accidents (22,241,242). N-hCR-bearing-genes that affect nitrogen metabolism include *CPS1*_3N-hCR_, regulating the first committed step of urea-cycle entry, and *SLC6A8*_3N-hCR_, a creatine transporter. Impaired translation of either gene could tend to cause ammonia toxicity due to urea cycle dysregulation. Indirect support for a link between elongation rate and altered mental status (*cf*. *KIF3C*_4N-hCR_) (243,244) comes from computational studies noting that *SHANK-2* and *SHANK-3*, but not *SHANK-1*, demonstrate traditional 'codon-use-bias', suggesting that a translational regulatory mechanism may underly *SHANK* mediated autism spectrum disorders (245). Since *SHANK* family genes are associated with schizophrenia and *SHANK-1, -2*, and -*3* are associated with autism, *SHANK1*3N-hCR could mediate mental status changes through altered translation rate that could be caused by fluctuations in plasma Asn concentrations.

Adverse neurological outcomes have also been associated with N-hCR-bearing-genes *ANK3*, *IRS2*, *SNCAIP*, *XIRP2*, *PPP1R9A* and *CACNA1-C*. Low plasma Asn, via the 17 N-hCR-bearing-genes listed in Fig. 1, can thus also plausibly be linked to onset of age associated(251) and *SNCAIP*; dental caries and peridontal disease as a diabetes comorbidity through *TMEM178B* or *ANKRD17* in children (252,253); (*cf*. *LRP1B* and periodontitis in adults) (254). Also affected by *LRP1B* are age at menarche (255), APOE and fibrinogen binding (256), protection from cognitive decline in aging (257) as well as BMI, insulin resistance, optic disc size/area (*cf*. glaucoma), conditional erectile dysfunction in African American men, heart rate and multiple cancers. Deafness (258,259) is affected by *XIRP2* (*cf*. *Xeplin*, *PTPRQ*), heroin addiction vulnerability in African Americans (260) and heart disease by *XIRP2* (261,262); heart disease by *PHACTR1* (263) (*cf*. *LRP6*) and *PPP1R9A* (*cf*. *CHRM-2, -3*) (264); bone density by *PHACTR1* (*cf*. *LRP4, LRP5*); erythropoesis and quality control of mitochondria by *BNIP3L*; nucleic acid processing by *COIL*, *PAPD5*, *THRAP3*, *MEX3B* and *C1orf86/FAAP20*; and diabetes by *THRAP3* (*cf*. *CHRM3*), *PTPRD* and *IRS2*.

### BNIP3L and PEG10: cancer and frameshifting

The discussion above has focused on adverse events elicited by ASNase therapy, not the induction of tumor remission. Two N-hCR-bearing-genes, *PEG10* and *BNIP3L*, have transcripts with long N-hCR that are encompassed within their initial two dozen codons. Both *BNIP3L* and *PEG10* are apoptosis-related genes that are candidates for mediation of the cell death that has been observed to follow depletion of Asn either in cell culture (265) or in pediatric ALL. Multiple other N-hCR-bearing-genes are also potential targets, e.g., *APC*, (*ARID5B*, *IL9R* and *RYR2*) (266), *JAK2*, *KCNA3* (145), *UBE2Q2* (267), *COIL* (268) or *SMG1*_2x3N-hCR_ (269) (a Ser-Thr kinase with homology to *mTOR*). Temperature sensitive mutants of Asn tRNA synthetase undergo cell cycle arrest in early S phase at the nonpermissive temperature, a phenomenon that has been posited to be consistent with the existence a protein required for cell cycle progression that is highly sensitive to the level of charged Asn-tRNA (270), such as one encoded by an N-hCR-bearing-gene that is eliminated and must be resynthesized once per cell cycle (*cf*. *COIL* above).

## 3. Evidence for and against the model, caveats

### In vitro translation and in vivo half lifes are consistent with ASNase impaired translocation at N-hCR

ASNase in *E. coli*, as well as in other gram negative bacteria (*Salmonella*, *Klebsiella*) (271), is encoded by two independent genes *AsnA* and *AsnB*. The *AsnB* product is periplasmic and is the therapeutic enzyme whereas the *AsnA* product is a cytoplasmic enzyme with a lower K*_m_* (272). Studies of a cytostatic factor produced by *Salmonella* led to its isolation and identification as ASNase, virtually identical to the *AsnB* product of *E. coli*. When added to *in vitro* translation extracts, it inhibited protein synthesis (16). To determine how it inhibited protein synthesis, i.e. if it simply depleted the levels of asparaginylated tRNAs available for translation, or if the process was more complicated (273,274) *in vitro* translation experiments (unpublished data) were performed with defined templates containing Asn codons at predetermined sites. T7 RNA polymerase was used to generate transcripts that were either devoid of Asn codons or contained one, two, five or 23 Asn codons between the N- and C-terminal segments of a bipartite hybrid protein composed of two human genes with no Asn codons. The N-terminal portion was derived from *TCL1A*, and the C-terminal portion was derived from *CKS2*. The central, intragenic N-hCR was, on occasion, substituted by the programmed ribosomal frameshifting (PRF) region from *PEG10* which contains an Asn (AAC) codon at the frameshifting site. The resulting *in vitro* transcripts were translated in rabbit reticulocyte cell free lysates with isotopically labelled ^35^S-methionine and the products were analyzed by sodium dodecyl sulfate (SDS)-polyacrylamide gel electrophoresis followed by autoradiography. This template gave extremely clean IVT results without the partial products seen with other templates such as *PEG10* or gaussia luciferase. It was determined, with some appropriate control experiments, that there were quantities of ASNase that could be added to the translation mix to create different ratios of partial to full length products which could reflect relative degrees of pausing at the different poly Asn regions of length zero, one, two, five and 23 codons. Free Asn could subsequently be added back to the depleted reaction mix to 'chase', to a first approximation, the short 'TCL1A' proteins into longer, hybrid, 'TCL1A/CKS2' proteins. Conditions were also established in which the relative efficiency of frameshifting at the Asn codon of the PRF site of PEG10 was affected by exogenous ASNase added to the *in vitro* translation reaction, but this result was far less compelling than the effect of ASNase on translocation at N-hCR.

We have seen full length translation of templates devoid of Asn codons under conditions of exogenously added ASNase, but in templates containing Asn codons, translated under identical conditions, we observe translation that extends to the N-hCR. Thus we suggest that depletion of Asn-ylated tRNA is likely to be the underlying cause of inhibition of synthesis seen previously by use of random, mixed templates for characterizing the inhibition, by *Salmonella* ASNase, of *in vitro* translation reactions (16). There were also unanticipated findings suggesting that frameshifting efficiency may depend on the number of Asn codons in an artificial N-hCR that was inserted a dozen codons upstream of the *PEG10* frameshifting site. We have not characterised the behavior of deamidated Asn-tRNA^Asn^ which could incorporate Asp residues at Asn codons were it not edited and removed by a proofreading complex.

### Differences in response to ASNase administration in children and adults, a recent gene family expansion

There are differences in response to ASNase between children and adults. They are most obvious in the ALL tumor remission response, as well as in the type of glycemic dysregulation: periphiral vs. central loss of responsiveness. In the pediatric patients, the hyperglycemia is insulin reversible, insulin is absent from circulation following an ASNase therapeutic regimen that includes steroid hormones similar to prednisolone, and it is likely that central control over insulin synthesis or release may be deficient. In the metabolomic studies of diabetic adults, Fasting Insulin levels are high, and IRS2 mediated peripheral signalling may be deficient. In addition, the unacceptable neurovascular complications (fugue state, cerebrovascular accidents) in adults compared to children underscores the difference between the physiology of children and adults.

The evolutionarily recent duplication of the *TBC1D3*_3N-hCR_ gene of hominids, and the expansion, and perhaps positive selection in humans, of eight members of this N-hCR bearing-gene family (275), suggests that these oncogenes (associated with ovarian cancer) (227) whose turnover is regulated by palmitoylation (276), may control vesicle fusion by nonca-nonical regulation of RAB GTP exchange (277), perhaps in association with Rab5 (278) [*cf*. TBC1D5 with Rab7 (279) or autophagy with ATG-8 (280) or ATG-9 (280)]. *TBC1D3* is involved in pinocytosis with ARF6 (281), affects epidermal growth factor receptor (EGFR) signalling by altering microtubule dynamics (282) and can influence insulin signalling (280) by regulating IRS1 degradation (284 *cf*. 285). These genes could also potentially regulate insulin or amino acid release from vesicular or lysosomal storage (286).

### AAC codons; intrinsically disordered protein assemblies

Most of the poly Asn codon runs reported here consist of the two isoaccepter codons AAT and AAC used in about equal frequency with a slight bias towards homopolymeric runs of AAC. In the gene *IRS2*_8N-hCR_, from human, zebrafish, elephant-shark, frog, python and falcon, AAC is used exclusively in N-hCR runs of varying length and distance from the initiator methionine, suggesting that if regulation is not restricted only to the AAC isoacceptor species, perhaps there is a further, structural, component to this phenomenon [CAG homocopolymers encoding poly Q repeats can form triple stranded structures (287), RNA sequences enriched in AAT motifs can be labile (288)]. Interestingly, *PEG10*7_N-hCR_ and *BNIP3L*_5N-hCR_ employ AAC codons exclusively in human and mouse (*PEG10*), or in human, mouse, rat, lizard, ~frog and chicken (*BNIP3L*), indicating that the two isoacceptor tRNAs may indeed be differentially regulated.

N-hCR-bearing-genes encode proteins that engage in networks whose equilibria may be affected by elongation rate, e.g. *PPP1R9A*_5Nx2-hCR_, unique among the 17 genes of Fig. 1 because of two separate N-hCR, encodes neurabin, the intrinsically disordered regions (289) of which become conformationally restricted in regulatory complexes with PP1 (290), and which is implicated in neurite formation (291), neuroprotection against seizures (292), mood disorders (293), hippocampal plasticity (294), long term depression (295), dopamine mediated plasticity (296), contextual fear memory (297), hepatosplenic lymphoma (298) and regulation of G protein coupled receptor (GPCR) signalling (250). A key unstructured UBZ domain of Fanconi's anemia gene *FAAP20* can form a highly structured α helix upon ubiquitin binding; this domain is interrupted by a 5N-hCR in certain variant isoforms. The 2N-hCR of TP53 is similarly located: adjacent to a pair of transactivation domains (TADs) that gain structure upon ligand binding (299,300). The N-hCR of *TRPM-6* and -*7* interrupt their α kinase domain. Modulating translation rate by varying Asn concentration, while synthesising these proteins, could allow modulation of the protein assemblies in which these proteins participate.

### Caveats, Asn residues can be post-translationally modified; interspecies N-hCR length variation and inflammation

In this survey of other potential roles for the conserved poly Asn regions in proteins, we note that they also act as sites of post-translational modification to regulate protein activity by glycosylation or deamination or [cleavage, by Asparaginyl endopeptidases (301) (*cf*. Taspase1, an ASNase gene family member) (302)]. The 4N-hCR of CFTR, differing in length between human, mouse and pig, encodes a conformationally dynamic regulatory insertion (303) that may gate access to the ATP binding site (304). A similarly unstructured loop in Bcl-xL undergoes deamination (305,306), as does an Asn residue pair between the TADs of TP53 (307), a region unstructured until bound to MDM2 (308,309). The 2N-hCR of TP53 differs in length between rats, mice and humans. N-hCR length variation in N-hCR-bearing-genes can correlate with disease severity in animal models of human inflamation. For example the pig model of CF more closely reflects the physiology of the human disorder, in comparison to the mouse model (310) perhaps because, as with TP53, the length of the poly Asn region in pig more closely resembles that of human rather than mouse. Also, in *P. aeruginosa*-induced bacteremic shock, TXNIP exacerbates septic shock associated with bacteremia in a mouse model (92). TXNIP of mouse has an identically situated, but longer poly Asn region (8N-hCR) than human and most other nonrodent mammals (3N-hCR), perhaps enabling greater redox level changes in response to Asn level variation. These examples may reflect divergent evolutionary choices in inflammatory and pathogen response strategies that may partially explain the reported differences between human and rodent models of inflammation (311,312) and IRS2 genetic associations (72). Altered electrophoretic mobility, a hallmark of some deamination events, indicates that post-translational modification may even occur at the poly Asn region of IRS (281). Deletion analysis of the N-terminal poly Asn containing region of *BNP3L/B5/NIX* suggests that it masks apoptosis inducing function (313,314). Regarding self association and aggregation at poly Asn regions, Perutz stated that it is unlikely that poly Asn repeats can form polar zippers of the kind formed by poly Gln repeats (315), but see (316). hCR may be tolerated at intrinsically disordered regions of proteins (317) where proteins could accommodate hCR expansion in their genes (318). An alternative explanation for the action of ASNase: NH3 generated by ASNase may act as a gaseous reactive signalling molecule, akin to NO, CO or SH2, to modify protein structure and function (319).

## 4. Biochemistry of amino acid activation, genome-wide association studies

At least five different human tRNA synthetases can serve as autoantigens in inflammatory responses (320). Human tRNA synthetases AsnRS and HisRS both serve as chemoattractants (321), ligands for cell surface proteins CCR5 and CCR3 respectively (322). AsnRS protein levels are upregulated by almost three orders of magnitude in a model of preosteoblast cell proliferation driven by FGF2 (323). Filarial AsnRS, in contrast to human AsnRS, serves as a ligand for CXCR1 and CXCR2 and is chemotactic for neutrophils and eosinophils, with a terminal subdomain that serves as a ligand for human IL8 receptor (324). The link between inflammatory responses and Asn tRNA synthetases remains an open question.

Leu contributes to formation of mTOR1C, a biochemical complex that regulates cell cycle (325) in conjunction with other amino acids (326,327) including Arg (328,329) and Gln (105,330–332). In a related experimental paradigm, apoptosis induced by Gln withdrawal, Asn, instead of Gln may actually be the effector molecule whose withdrawal is sensed (267). A biochemical mechanism for sensing Asn levels, required either to trigger apoptosis, or to advance through S phase of the cell cycle, perhaps mediated by AsnRS, and not involving ribosomes may yet be discovered, but even if such a mechanism were to exist, translational inhibition at N-hCR would still remain a most parsimonious explanation for the myriad clinical side-effects of ASNase treatment. Poly Asn (2) and poly Leu (100) codon repeats (N-hCR and L-hCR) appear in a biased manner in mammalian genomes; this bias may be related to metabolomic differences in the levels of Asn (23,28) and Leu (333) between normal and diabetic patients as we have discussed for the case of Asn in this study, and as may be the case for Leu (*cf*. L-hCR length polymorphisms and diabetic nephropathy in *CNDP1* (107,108). mTORC1 activation is the orthodox pathway for understanding how altered amino acid levels exert metabolic control. This study has examined an alternative hypothesis, of the potential for amino acid fluctuations to control translation rate, to thereby effect a different measure of metabolic control by reshaping the composition of the proteome.

### Genome-wide association studies (GWAS)

GWAS have met limited success (190,334–336). The contribution of the environment to gene expression is particularly difficult to quantify but it may explain the missing heritability problem (337). The biomic environment has a significant impact on gene expression, and part of its function could be to alter levels of plasma amino acids that may ultimately be reflected in intracellular amino acid level variation and alterations in translation rates within those cells. If the genomic bias in N-hCR use is a harbinger of a broad effect of inhibited translation due to Asn level variation, then GWAS screens for common disorders may reveal N-hCR-bearing-genes that could be influenced by constituents of the biome that alter Asn concentrations and could contribute to metabolism, aging and complex diseases.

GWAS of five major psychiatric illnesses implicates four N-hCR-bearing-genes (338). Most prominent is *ANK3* (one of the top 17 N-hCR-bearing-genes) (*cf*. Fig. 1) as well as *CACNA1C*, *ZFPM2* and *NTRK3*. *NTRK3* can be related, through a neuronal cell death mechanism (339), to *mBEX3* (340), a murine gene that bears a long N-hCR. *NTRK3* is associated with Gaucher's disease, PD (341,342), multiple cancers (343–347) leukemia (348), and is an entry receptor for trypanosomes (349) (*cf*. *APOL1, PTPRD, PHACTR1*) (350). Asn level variation may affect all of these processes. In a GWAS of seven common diseases, hypertension was most closely associated with two linked N-hCR-bearing-genes, *RYR2* and *CHRM3*. *RYR2* is involved with heart disease (351) and associated with lipid levels (352) and ALL (266), *CHRM3*_3N-hCR_ is associated with response to an antidiabetic drug in African Americans (353) (*cf*. *CHRM2*_4N-hCR_ associated with metabolic syndrome) (354). Another of the seven common diseases, Crohn's disease, was quite significantly associated with an N-hCR-bearing-gene, *IL23R* (355). *IL23R* is also associated with psoriasis, diabetes (356), CAD, Behcet's disease, ankylosing spondylitis (357–359) and leprosy (360).

A GWAS of ALL shows that it is affected by at least two other N-hCR-bearing-genes, in addition to *RYR2* (noted above): *IL9R* (361) and *ARID5B* (*cf*. *KCNA3*) (145). *IL9R* shares a common γ subunit with other interleukin receptors) (362) *IL9R* has a 4N-hCR that is absent from all mammals except *Pan* [*cf*. *APOL1* which lacks 3N-hCR in all mammals except *Gorilla* (2N in *Pongo*)]. *ARID5B* encodes part of a histone lysine demethylase complex (363) and is not only genetically associated with ALL (266,364–369) but is also associated with corneal changes (370), low birth weight (371), diastolic blood pressure (372) rheumatoid arthritis (373), response to haloperidol (374) (an anti-psychotic medication), systemic lupus erythematosus (SLE) (375), lipid balance (376) and triglyceride metabolism in mouse adipocytes (377), as well as, in humans, T2DM (378). The contribution of ASNase to these conditions, especially to ALL, potentially by altered translation at the N-hCR of *ARID5B* warrants further investigation (379).

We propose that the impaired translation which has been described above be termed the 'translational N-hamper effect' because there is nothing intrinsically impaired about a protein polymerization reaction in which one of the required components, activated Asn tRNA, is ratelimiting for the translocation reaction on the template mRNA. The verb of choice for slowed translocation could just as well have been cumbered movement instead of hampered movement. If the argument was first made for Gln, the Q-cumber effect could have encompassed this hypothetical phenomenon.

The 'translational N-hamper effect' is a mechanism whereby protein expression is modulated by coupling fluctuations in appropriate aminoacylated-tRNA availability to ribosome translocation rates at corresponding hCR. Thus, ribosome movement could pause at hCR which would serve as punctuation marks to allow relative intracellular amino acid pool sizes to influence mRNA decoding and protein synthesis. Amino acid level fluctuation could potentially affect: mRNA halflife and accessibility to regulatory complexes, ribosome frameshifting efficiency, initiation rate and formation of stable translation complexes, and elongation rate and vestibule residence time to affect steady state levels of these proteins and of higher order structures in which they participate.

Our model holds that Asn level reductions, such as those accompanying the administration of ASNase, cause impaired translation of N-hCR-bearing-genes to precipitate metabolic, vascular, immunological and neurological disorders and contends that this could result in insulin desensitization, impaired insulin release and, ultimately, diabetes. Thus the microbiome, by endogenously generating ASNase, could cotranslationally regulate a constellation of N-hCR-bearing-genes to initiate complex disease pathologies.

## Figures and Tables

**Table I:** Alphabetical listing of 765 human genes 3N-hCR and higher (>3N-hCR).

*A2M*	*ATP6V1C1*	*CES2*	*DNAH1*	*FSIP2*	*KHDRBS2*	*LY75*
*AATK*	*BAG5*	*CFAP45*	*DNAH6*	*FSTL3*	*KIAA0232*	*LYST*
*ACACA*	*BAG6*	*CFAP54*	*DNAJB11*	*G3BP1*	*KIAA1024L*	*MALT1*
*ACACB*	*BAI2*	*CFTR*	*DNAL4*	*GABBR1*	*KIAA1107*	*MAML2*
*ACAN*	*BCAS1*	*CGRRF1*	*DNM1L*	*GBP6*	*KIAA1210*	*MAP7*
*ACSBG2*	*BCAS3*	*CHAD*	*DNMT3A*	*GCLC*	*KIAA1217*	*MAPK8IP2*
*ADAM10*	*BIN2*	*CHD7*	*DNTTIP2*	*GDPD1*	*KIAA1549L*	*MAPRE2*
*ADAM19*	*BIRC6*	*CHEK2*	*DOCK4*	*GGA1*	*KIAA1586*	*MARCH1*
*ADAM30*	*BMPR2*	*CHFR*	*DRD1*	*GGA3*	*KIAA1671*	*MARCH6*
*ADCY8*	***BNIP3L***	*CHRM2*	*DSCAM*	*GIN1*	*KIAA1841*	*MASP1*
*ADCY9*	*BOC*	*CHRM3*	*DSPP*	*GIT2*	*KIDINS220*	*MBD5*
*AEBP1*	*BOD1L1*	*CHRNB1*	*DUSP10*	*GJA9*	*KIF16B*	*MDGA2*
*AFF2*	*BRIP1*	*CHRND*	*DUSP21*	*GK*	*KIF1A*	*MED1*
*AGAP1*	*BRCA2*	*CHSY1*	*DYNC1H1*	*GKN1*	*KIF21A*	***MEX3B***
*AGBL2*	*BTAF1*	*CKAP2L*	*DYNC1I1*	*GNAZ*	*KIF3C*	*MGAM*
*AKAP4*	*BTBD1*	*CLCA1*	*DYNC1I2*	*GNPAT*	*KLF17*	*MGAM2*
*ALDH6A1*	*BTBD2*	*CLCA2*	*DYRK4*	*GOLPH3*	*KLHL3*	*MGAT2*
*ALKBH8*	*BTBD3*	*CLCA3P*	*DZIP1*	*GP1BA*	*KLHL30*	*MIB1*
*ALPK2*	*BTG4*	*CLCA4*	*ECM2*	*GPATCH2*	*KMT2A*	*MID1*
***ALS2CR11***	*C18orf63*	*CLEC10A*	*EFNB2*	*GPR112*	*KMT2E*	*MIS18BP1*
*AMBRA1*	***C1orf86***	*CLEC6A*	*EIF2A*	*GPR126*	*KNG1*	*MITF*
*AMY2A*	*C1QB*	*CLMN*	*ELAVL2*	*GPR64*	*l101060321*	*MLLT3*
*AMY2B*	*C1QL2*	*CLTC*	*ELF1*	*GPR82*	*l101060389*	*MON2*
*ANAPC7*	*C1QL3*	*CNOT10*	*EOMES*	*GSG2*	*l102723859*	*MTBP*
***ANK3***	*C2orf49*	*CNOT2*	*EPCAM*	*GTF2I*	*l102724862*	*MTCH1*
*ANKFN1*	*C2orf61*	*CNOT6*	*EPPIN*	*HACL1*	*l102725117*	*MTERF1*
*ANKFY1*	*C3*	*CNOT6L*	*EPPIN-WFDC6*	*HAVCR1*	*LAMA3*	*MTG2*
*ANKRD17*	*C3orf67*	*CNST*	*EPRS*	*HCFC2*	*LAMB4*	*MTNR1A*
*ANKRD28*	*C5orf67*	*COBL*	*EPYC*	*HECTD4*	*LAMC2*	*MTTP*
*ANKRD44*	*C7*	*COBLL1*	*EVI2A*	*HERC6*	*LAMP2*	*MTUS2*
*ANKRD7*	*CACHD1*	***COIL***	*EYA1*	*HERPUD1*	*LARP4*	*MUC19*
*ANPEP*	*CACNA1A*	*COL24A1*	*F5*	*HERPUD2*	*LEMD3*	*MUC3A*
*ANTXR1*	*CACNA1C*	*COL6A2*	*FAM117B*	*HLA-DPA1*	*LGI1*	*MUC4*
*ANTXRL*	*CACNA1D*	*COL6A5*	*FAM126A*	*HLTF*	*LGI3*	*MXRA5*
*AP2B1*	*CACNA1F*	*COX19*	*FAM171B*	*HMCN1*	*LGR6*	*MYO10*
*AP4E1*	*CACNA1H*	*CPEB4*	FAM193A	*HNRNPL*	*LIMS2*	*MYO19*
*APBA2*	*CACNA1S*	*CPM*	*FAM208B*	*HNRNPUL1*	*LINGO2*	*MYO1A*
*APC*	*CALHM1*	*CPNE9*	*FAM65B*	*HRG*	*LITAF*	*MYO1B*
*APCDD1*	*CARF*	*CPS1*	*FAM69C*	*HSD3B1*	*LPHN2*	*MYO1E*
*APOB*	*CASC5*	*CPXM2*	*FANCI*	*HSPG2*	*LRFN2*	*MYO1F*
*APOL1*	*CASS4*	*CRTAC1*	*FAT2*	*HYPM*	*LRFN5*	*MYO6*
*AQP5*	*CASZ1*	*CSMD2*	*FAT3*	*ICE1*	*LRIG1*	*MYO9A*
*ARHGAP11A*	*CATSPERD*	*CSTF3*	*FAT4*	*IGDCC3*	*LRIG2*	*MYO9B*
*ARHGAP20*	*CCDC144A*	*CUL1*	*FBXL5*	*IGLV10-54*	*LRIG3*	*MYOM1*
*ARHGAP24*	*CCDC144NL*	*CUL3*	*FBXO27*	*IL1RAP*	*LRP1B*	*MYRF*
*ARHGEF10*	*CCDC18*	*CXCL12*	*FBXO38*	*IL23R*	*LRP2*	*MYT1L*
*ARHGEF5*	*CCDC36*	*CYP19A1*	*FBXO39*	*IL9R*	*LRP4*	*N4BP2*
*ARHGEF6*	*CCDC39*	*CYP1A1*	*FBXO48*	*ING3*	*LRP5*	*NBN*
*ARID1A*	*CCDC73*	*CYSLTR2*	*FBXO5*	*INTS12*	*LRP6*	*NBR1*
*ARID1B*	*CCDC88A*	*DCAF6*	*FBXW7*	*IPMK*	*LRPPRC*	*NCAM2*
*ARID5B*	*CCKAR*	*DCAF7*	*FCGR2A*	*IRAK3*	*LRRC30*	*NCAPH2*
*ARMC3*	*CCNT1*	*DCBLD1*	*FCGR2B*	*IRS2*	*LRRC37A*	*NCKAP1*
*ARMC4*	*CD63*	*DCN*	*FCGR2C*	*ISLR2*	*LRRC37A2*	*NCOA1*
*ARPP21*	*CD9*	*DDIAS*	*FCN1*	*ITGAV*	*LRRC37A3*	*NCOA3*
*ASB2*	*CDC14A*	*DDR2*	*FCRL4*	*ITGB1BP1*	*LRRC38*	*ND4*
*ASCL5*	*CDH9*	*DDX4*	*FEZ1*	*ITK*	*LRRC57*	*NECAB3*
*ASIC2*	*CDHR1*	*DDX42*	*FGB*	*JAK2*	*LRRC69*	*NEDD1*
*ASPN*	*CDKL5*	*DDX59*	*FKBP7*	*JMJD1C*	*LRRC70*	*NEURL4*
*ATAD5*	*CDON*	*DHX38*	*FLII*	*JMY*	*LRRC71*	*NFATC1*
*ATF7IP*	*CEACAM5*	*DIAPH1*	*FLRT1*	*KCNA3*	*LRRC72*	*NGLY1*
*ATF7IP2*	*CELSR3*	*DIDO1*	*FLRT3*	*KCNH4*	*LRRC8B*	*NIPA2*
*ATL2*	*CEMIP*	*DLGAP5*	*FNDC4*	*KCNH8*	*LRRN1*	*NKX2-5*
*ATP2B1*	*CENPC*	*DMD*	*FNDC5*	*KDM3A*	*LRRN2*	*NNT*
*ATP2B3*	*CEP350*	*DMXL2*	*FRS3*	*KDM6A*	*LRTOMT*	*NOD1*
*ATP2B4*	*CERS2*	*DMKN*	*FSHR*	*KDM6B*	*LTF*	*NOS2*
*NOTCH1*	*PKDREJ*	*RDH10*	*SLC2A12*	*SUSD1*	*TMEM259*	*UTY*
*NPNT*	*PKD1L3*	*REG4*	*SLC35A4*	*SUZ12*	*TMOD1*	*VEPH1*
*NPY1R*	*PKHD1L1*	*RELA*	*SLC6A11*	*SYCP1*	*TMPRSS11A*	***VEZF1***
*NPY6R*	*PKP1*	*RGL1*	*SLC6A4*	*SYNPO2*	*TMPRSS11D*	*VGLL4*
*NR1D1*	*PLEKHG3*	*RLF*	*SLC6A8*	*TAB2*	*TMPRSS15*	*VN1R2*
*NRK*	*PLS1*	*RMI1*	*SLCO3A1*	*TAB3*	*TNRC6A*	*VPS13A*
*NRP1*	*PMS1*	*RNF103*	*SLIT1*	*TALPID3*	*TNRC6B*	*VPS4A*
*NSUN7*	*PNLIPRP1*	*RNF128*	*SLIT2*	*TANGO2*	*TOX3*	*VPS45*
*NT5E*	*POGZ*	*RNF139*	*SLIT3*	*TAS2R38*	*TPGS1*	*WDR13*
*NTRK3*	*PPAP2B*	*RNF157*	*SLITRK1*	*TAX1BP1*	*TPRKB*	*WDR17*
*NUP54*	*PPP1R13B*	*RNF180*	*SLITRK2*	*TBC1D3*	*TRAJ31*	*WDR48*
*OBSL1*	*PPP1R36*	*RNF19A*	*SLITRK3*	*TBC1D3B*	*TRAJ39*	*WFDC6*
*OGG1*	*PPP1R3A*	*RNF2*	*SLITRK4*	*TBC1D3C*	*TRAJ43*	***XIRP2***
*OIT3*	*PPP1R42*	*RNF213*	*SLITRK5*	*TBC1D3F*	*TRAPPC12*	*YAE1D1*
*OLFM4*	*PPP1R7*	*RNF216*	*SLITRK6*	*TBC1D3H*	*TRIP12*	*ZAN*
*OMG*	***PPP1R9A***	*RNF220*	*SMARCA2*	*TBC1D3K*	*TRPM6*	*ZBTB10*
*OR4A5*	*PPP3CB*	*ROBO2*	*SMARCA4*	*TBC1D3L*	*TRPM7*	*ZBTB6*
*OR4C16*	*PPP3CC*	*RP1*	*SMG1*	*TBC1D5*	*TSC22D3*	*ZC3HAV1*
*OR8G5*	*PRDM12*	*RPGR*	*SNAP91*	*TBR1*	*TSEN2*	*ZCCHC11*
*OSCP1*	*PRDM2*	*RUSC1*	***SNCAIP***	*TCHHL1*	*TSHZ3*	*Z FAND3*
*OTOG*	*PRELP*	*RYR2*	*SNED1*	*TCN1*	*TSPAN17*	*ZFP1*
*OVGP1*	*PREX1*	*RYR3*	*SNRPA1*	*TCTN2*	*TSPAN5*	*ZFPM2*
*P2RY10*	*PRF1*	*S100PBP*	*SOCS4*	*TEC*	*TSPYL2*	*ZFYVE1*
*PAN3*	*PRL*	*SALL4*	*SON*	*TECTA*	*TTC1*	*ZFYVE28*
***PAPD5***	*PSMD1*	*SCARB1*	*SOWAHD*	*TEKT1*	*TTC8*	*ZIC4*
***PAPPA-AS1***	*PSMD3*	*SCP2*	*SP4*	*TENM3*	*TTLL4*	*ZMIZ2*
*PARG*	*PSMF1*	*SCRN3*	*S PA TA16*	*TENM4*	*TTLL5*	*ZMYM6*
*PARP2*	*PTPRB*	*SDAD1*	*SPDYA*	*TESK1*	*TXLNG*	*ZNF132*
*PARP3*	*PTPRD*	*SEC16A*	*SPECC1*	*TEX15*	*TXNIP*	*ZNF23*
*PAW R*	*PTPRQ*	*SEC24B*	*SPRY1*	*TEX2*	*TXNL4A*	*ZNF236*
*PCDH7*	*PUM1*	*SEZ6L2*	*SPSB1*	*THEG*	*UBAC1*	*ZNF347*
*PCDHAC2*	*PXDN*	*SGOL2*	*SPTBN4*	***THRAP3***	*UBE2Q2*	*ZNF451*
*PCDHGA3*	*PXDNL*	*SH3BP5*	*SRPRB*	*THSD7B*	*UBE4A*	*ZNF518A*
*PCSK2*	*PXMP4*	*SH3D19*	*SSH1*	*TINAGL1*	*UBXN7*	*ZNF804A*
*PDE3A*	*PYGO1*	*SH3GLB1*	*S TAB2*	*TKT*	*ULK4*	*ZNRF3*
*PEAK1*	*PZP*	*SHANK1*	*S TAU2*	*TLR10*	*URB2*	*ZPLD1*
***PEG10***	*QSER1*	*SHCBP1L*	*STK32A*	*TLR2*	*USO1*	*ZXDA*
*PFKFB2*	*R3HDM2*	*SHOC2*	*STK32B*	*TLR3*	*USP11*	*ZXDB*
*PGBD2*	*RAB3GAP1*	*SI*	*STMN1*	*TM4SF18*	*USP12*	*ZZEF1*
***PHACTR1***	*RANBP17*	*SIN3A*	*STMN2*	*TMCO1*	*USP13*	*ZZZ3*
*PHF2*	*RAPGEF2*	*SIN3B*	*STMN3*	*TMCO2*	*USP26*	
*PIK3CB*	*RBM12*	*SIPA1L1*	*STMN4*	*TMEM106B*	*USP31*	
*PIK3R1*	*RBM27*	*SIX1*	*SULF1*	***TMEM178B***	*USP32*	
*PJA1*	*RBM28*	*SLC18A1*	*SULF2*	*TMEM2*	*USP34*	
*PJA2*	*RBMS1*	*SLC26A9*	*SUMO4*	*TMEM57*	*UTRN*	

The top 17 listed on Fig. 1 from 8N-hCR to 5N-hCR are in bold font; the 49 genes with 4N-hCR are underlined. A total of 699 genes on this list have 3N-hCR and are in normal font (not bold or underlined). **17×5N-hCR**, 49**×**4N-hCR, 699**×**3N-hCR. Each N-hCR-bearing-gene and its corresponding protein in the NCBI homologene database, were used in this analysis except for the following 28 genes: *APOL1* isfX1, X2; *ANKRD28* iCRA_g; *C1orf86/FAAP20* tvi4X1,2,3; *DMKN* i5; *FBXO38* iCRA_d; *FKBP7* isf23 AF100751.1; *IGLV10-54* BAA19993.1; *KHDRBS2* iCRA_c; *loc102725117* isf.X1-7; *LRTOMT* isf1c,1a; *MARCH1* ix1; *MASP1* isf1; *MGAM* int iX1; *MTCH1* AAD34059.1; *NTRK3* isof ×10, XP_006720612; *PAAAA-AS1* AAV41520.1; *PTPRB* iX5; *PHACTR1* iX6; *RAPGEF2* iX7; *RNF128* isf2; *SH3D19* isfX2,4,5,6,8; *SNAP91* isfD; *TRAJ31,39,43* AAB86765.1, AAB86758.1, AAB86754.1; *VEZF1* iCRA_a,c; *WFDC6* iCRA_a,b; *WDR17* iX5; *XIRP2* tv5 and tv3; *ZFP1* iX1. A number of N-hCR-bearing-genes are in GTPase, GPCR or odorant receptor families, or can be grouped as involved with ubiquitin conjugation, DNA repair, RNA processing,or pattern recognition response. The relative frequency of appearance of such genes among the N-hCR-bearing genes versus their proportional representation in the human genome remains uncharacterized. CKS2, on a list of genes that are devoid of Asn codons in mammalia, is a paralog of a plasmodium protein (XP_001352106) which has the longest contiguous stretch of 83 Asn residues in plasmodia (382,383). When the plasmodium gene is compared to the human database, the best 3 homologies are to *CKS2, CKS1B* and the N-hCR-bearing-gene *PPP1R13B_4N_*_-hCR_/*ASPP1*, the promoter of which is silenced by methylation in ALL (384). The balance between *CKS2* and *CKS1B* is thought to play a role in multiple cancers (385), including HHV4 associated nasopharyngeal cancer (386) (along with TRPM74N-hCR). Altered Asn levels could shift the balance between CKS2 and CKS1B to affect cell cycle regulation in multiple cancers including ALL, and, via PPP1R13B, senescence in normal cells (387). Other notable genes devoid of Asn codons are mus *APRT* (kidney stones) (388) and human *BIRC7* (ALL prognosis) (389), *LOR* (*cf*. *Staph.* aureous infection of nares) (390), *SEPW1* (cell cycle) (391), *TCL1* (leukemia) (392), *CSF3* (innate immunity and aneurysms) (393) and *KLF16* (proposed master metabolic regulator *KLF14*) (394).

## References

[1] Karlin S, Brocchieri L, Bergman A, Mrazek J, Gentles AJ (2002). Amino acid runs in eukaryotic proteomes and disease associations. Proc Natl Acad Sci U S A.

[2] Kreil DP, Kreil G (2000). Asparagine repeats are rare in mammalian proteins. Trends Biochem Sci.

[3] Karlin S, Burge C (1996). Trinucleotide repeats and long homopeptides in genes and proteins associated with nervous system disease and development. Proc Natl Acad Sci USA.

[4] Kawedia JD, Rytting ME (2014). Asparaginase in acute lymphoblastic leukemia. Clin Lymphoma Myeloma Leuk.

[5] Müller HJ, Boos J (1998). Use of L-Asparaginase in childhood ALL. Crit Rev Oncol Hematol.

[6] Suzuki R (2014). Pathogenesis and treatment of extranodal natural killer/T-cell lymphoma. Semin Hematol.

[7] Fréling E, Granel-Brocard F, Serrier C, Ortonne N, Barbaud A, Schmutz J (2015). Extranodal NK/T-cell lymphoma, nasal-type, revealed by cutaneous breast involvement. Ann Dermatol Venereol.

[8] Kidd JG (1953). Regression of transplanted lymphomas induced in vivo by means of normal guinea pig serum. I. Course of transplanted cancers of various kinds in mice and rats given guinea pig serum, horse serum, or rabbit serum. J Exp Med.

[9] Broome JD (1963). Evidence that the L-asparaginase of guinea pig serum is responsible for its antilymphoma effects. I. Properties of the L-asparaginase of guinea pig serum in relation to those of the antilymphoma substance. J Exp Med.

[10] Essig S, Li Q, Chen Y, Hitzler J, Leisenring W, Greenberg M, Sklar C, Hudson MM, Armstrong GT, Krull KR (2014). Risk of late effects of treatment in children newly diagnosed with standard-risk acute lymphoblastic leukaemia: A report from the Childhood Cancer Survivor Study cohort. Lancet Oncol.

[11] Tong WH, Pieters R, Hop WC, Lanvers-Kaminsky C, Boos J, van der Sluis IM (2013). No evidence of increased asparagine levels in the bone marrow of patients with acute lymphoblastic leukemia during asparaginase therapy. Pediatr Blood Cancer.

[12] Fine BM, Kaspers GJ, Ho M, Loonen AH, Boxer LM (2005). A genome-wide view of the in vitro response to l-asparaginase in acute lymphoblastic leukemia. Cancer Res.

[13] Kelo E, Noronkoski T, Stoineva IB, Petkov DD, Mononen I (2002). Beta-aspartylpeptides as substrates of L-asparaginases from Escherichia coli and Erwinia chrysanthemi. FEBS Lett.

[14] Chan WK, Lorenzi PL, Anishkin A, Purwaha P, Rogers DM, Sukharev S, Rempe SB, Weinstein JN (2014). The glutaminase activity of L-asparaginase is not required for anticancer activity against ASNS-negative cells. Blood.

[15] Huang L, Liu Y, Sun Y, Yan Q, Jiang Z (2014). Biochemical characterization of a novel L-Asparaginase with low glutaminase activity from Rhizomucor miehei and its application in food safety and leukemia treatment. Appl Environ Microbiol.

[16] Iwamaru Y, Miyake M, Arii J, Tanabe Y, Noda M (2001). An inhibitory factor for cell-free protein synthesis from Salmonella enteritidis exhibits cytopathic activity against Chinese hamster ovary cells. Microb Pathog.

[17] Capizzi RL, Bertino JR, Skeel RT, Creasey WA, Zanes R, Olayon C, Peterson RG, Handschumacher RE (1971). L-asparaginase: Clinical, biochemical, pharmacological, and immunological studies. Ann Intern Med.

[18] Bettigole RE, Himelstein ES, Oettgen HF, Clifford GO (1970). Hypofibrinogenemia due to L-asparaginase: Studies of fibrinogen survival using autologous 131-I-fibrinogen. Blood.

[19] Avramis VI (2014). Is glutamine depletion needed in ALL disease?. Blood.

[20] Quintanilla-Flores DL, Flores-Caballero MÁ, Rodríguez-Gutiérrez R, Tamez-Pérez HE, González-González JG (2014). Acute pancreatitis and diabetic ketoacidosis following L-asparaginase/prednisonetherapy in acute lymphoblastic leukemia. Case Rep Oncol Med.

[21] Frankel DL, Wells H, Fillios LC (1973). Concentrations of asparagine in tissues of prepubertal rats after enzymic or dietary depletion of asparagine. Biochem J.

[22] Holcenberg JS, Tang E, Dolowy WC (1975). Effect of Acinetobacter glutaminase-asparaginase treatment on free amino acids in mouse tissues. Cancer Res.

[23] Cheng S, Rhee EP, Larson MG, Lewis GD, McCabe EL, Shen D, Palma MJ, Roberts LD, Dejam A, Souza AL (2012). Metabolite profiling identifies pathways associated with metabolic risk in humans. Circulation.

[24] Kullas AL, McClelland M, Yang HJ, Tam JW, Torres A, Porwollik S, Mena P, McPhee JB, Bogomolnaya L, Andrews-Polymenis H, van der Velden AW (2012). L-asparaginase II produced by Salmonella typhimurium inhibits T cell responses and mediates virulence. Cell Host Microbe.

[25] Lavine RL, DiCinto DM (1984). L-asparaginase diabetes mellitus in rabbits: Differing effects of two different schedules of L-asparaginase administration. Horm Metab Res.

[26] Khan A, Adachi M, Hill JM (1969). Diabetogenic effect of L-asparaginase. J Clin Endocrinol Metab.

[27] Khan A, Adachi M, Hill JM (1970). Potentiation of diabetogenic effect of L-asparaginase by prednisolone. Horm Metab Res.

[28] Zhou Y, Qiu L, Xiao Q, Wang Y, Meng X, Xu R, Wang S, Na R (2013). Obesity and diabetes related plasma amino acid alterations. Clin Biochem.

[29] Nakamura H, Jinzu H, Nagao K, Noguchi Y, Shimba N, Miyano H, Watanabe T, Iseki K (2014). Plasma amino acid profiles are associated with insulin, C-peptide and adiponectin levels in type 2 diabetic patients. Nutr Diabetes.

[30] Burén J, Liu HX, Lauritz J, Eriksson JW (2003). High glucose and insulin in combination cause insulin receptor substrate-1 and -2 depletion and protein kinase B desensitisation in primary cultured rat adipocytes: possible implications for insulin resistance in type 2 diabetes. Eur J Endocrinol.

[31] Tsunekawa S, Demozay D, Briaud I, McCuaig J, Accili D, Stein R, Rhodes CJ (2011). FoxO feedback control of basal IRS-2 expression in pancreatic β-cells is distinct from that in hepatocytes. Diabetes.

[32] Argetsinger LS, Norstedt G, Billestrup N, White MF, Carter-Su C (1996). Growth hormone, interferon-gamma, and leukemia inhibitory factor utilize insulin receptor substrate-2 in intracellular signaling. J Biol Chem.

[33] Uddin S, Fish EN, Sher D, Gardziola C, Colamonici OR, Kellum M, Pitha PM, White MF, Platanias LC (1997). The IRS-pathway operates distinctively from the Stat-pathway in hematopoietic cells and transduces common and distinct signals during engagement of the insulin or interferon-alpha receptors. Blood.

[34] O'Connor JC, Sherry CL, Guest CB, Freund GG (2007). Type 2 diabetes impairs insulin receptor substrate-2-mediated phosphatidylinositol 3-kinase activity in primary macrophages to induce a state of cytokine resistance to IL-4 in association with overexpression of suppressor of cytokine signaling-3. J Immunol.

[35] Carey GB, Semenova E, Qi X, Keegan AD (2007). IL-4 protects the B-cell lymphoma cell line CH31 from anti-IgM-induced growth arrest and apoptosis: Contribution of the PI-3 kinase/AKT pathway. Cell Res.

[36] Blaeser F, Bryce PJ, Ho N, Raman V, Dedeoglu F, Donaldson DD, Geha RS, Oettgen HC, Chatila TA (2003). Targeted inactivation of the IL-4 receptor alpha chain I4R motif promotes allergic airway inflammation. J Exp Med.

[37] Wurster AL, Withers DJ, Uchida T, White MF, Grusby MJ (2002). Stat6 and IRS-2 cooperate in interleukin 4 (IL-4)-induced proliferation and differentiation but are dispensable for IL-4-dependent rescue from apoptosis. Mol Cell Biol.

[38] Butte NF, Voruganti VS, Cole SA, Haack K, Comuzzie AG, Muzny DM, Wheeler DA, Chang K, Hawes A, Gibbs RA (2011). Resequencing of IRS2 reveals rare variants for obesity but not fasting glucose homeostasis in Hispanic children. Physiol Genomics.

[39] Haghani K, Bakhtiyari S (2012). The study on the relationship between IRS-1 Gly972Arg and IRS-2 Gly1057Asp polymorphisms and type 2 diabetes in the Kurdish ethnic group in West Iran. Genet Test Mol Biomarkers.

[40] Ayaz L, Karakaş Çelik S, Cayan F (2014). The G1057D polymorphism of insulin receptor substrate-2 associated with gestational diabetes mellitus. Gynecol Endocrinol.

[41] Pezzolesi MG, Poznik GD, Skupien J, Smiles AM, Mychaleckyj JC, Rich SS, Warram JH, Krolewski AS (2011). An intergenic region on chromosome 13q33.3 is associated with the susceptibility to kidney disease in type 1 and 2 diabetes. Kidney Int.

[42] Craig DW, Millis MP, DiStefano JK (2009). Genome-wide SNP genotyping study using pooled DNA to identify candidate markers mediating susceptibility to end-stage renal disease attributed to Type 1 diabetes. Diabet Med.

[43] Kim SK, Yu GI, Park HJ, Kim YJ, Kim JW, Baik HH, Chung JH (2013). A polymorphism (rs4773092, Cys816Cys) of IRS2 affects auditory hallucinations in schizophrenia patients. Psychiatry Res.

[44] Acevedo N, Mercado D, Vergara C, Sánchez J, Kennedy MW, Jiménez S, Fernández AM, Gutiérrez M, Puerta L, Caraballo L (2009). Association between total immunoglobulin E and antibody responses to naturally acquired Ascaris lumbricoides infection and polymorphisms of immune system-related LIG4, TNFSF13B and IRS2 genes. Clin Exp Immunol.

[45] Alvarez-Perez JC, Rosa TC, Casinelli GP, Valle SR, Lakshmipathi J, Rosselot C, Rausell-Palamos F, Vasavada RC, García-Ocaña A (2014). Hepatocyte growth factor ameliorates hyperglycemia and corrects β-cell mass in IRS2-deficient mice. Mol Endocrinol.

[46] Withers DJ, Gutierrez JS, Towery H, Burks DJ, Ren JM, Previs S, Zhang Y, Bernal D, Pons S, Shulman GI (1998). Disruption of IRS-2 causes type 2 diabetes in mice. Nature.

[47] Niessen M (2006). On the role of IRS2 in the regulation of functional beta-cell mass. Arch Physiol Biochem.

[48] Park S, Hong SM, Lee JE, Sung SR, Kim SH (2008). Chlorpromazine attenuates pancreatic beta-cell function and mass through IRS2 degradation, while exercise partially reverses the attenuation. J Psychopharmacol.

[49] Gunasekaran U, Hudgens CW, Wright BT, Maulis MF, Gannon M (2012). Differential regulation of embryonic and adult β cell replication. Cell Cycle.

[50] Oliveira JM, Rebuffat SA, Gasa R, Gomis R (2014). Targeting type 2 diabetes: Lessons from a knockout model of insulin receptor substrate 2. Can J Physiol Pharmacol.

[51] Rametta R, Mozzi E, Dongiovanni P, Motta BM, Milano M, Roviaro G, Fargion S, Valenti L (2013). Increased insulin receptor substrate 2 expression is associated with steatohepatitis and altered lipid metabolism in obese subjects. Int J Obes (Lond).

[52] Minchenko DO, Davydov VV, Budreiko OA, Moliavko OS, Kulieshova DK, Tiazhka OV, Minchenko OH (2015). The expression of CCN2, IQSEC, RSPO1, DNAJC15, RIPK2, IL13RA2, IRS1, and IRS2 genes in blood of obese boys with insulin resistance. Fiziol Zh.

[53] Chen GT, Inouye M (1994). Role of the AGA/AGG codons, the rarest codons in global gene expression in Escherichia coli. Genes Dev.

[54] Mitarai N, Sneppen K, Pedersen S (2008). Ribosome collisions and translation efficiency: Optimization by codon usage and mRNA destabilization. J Mol Biol.

[55] Zhang S, Goldman E, Zubay G (1994). Clustering of low usage codons and ribosome movement. J Theor Biol.

[56] Chen GF, Inouye M (1990). Suppression of the negative effect of minor arginine codons on gene expression; preferential usage of minor codons within the first 25 codons of the Escherichia coli genes. Nucleic Acids Res.

[57] Ivanov IG, Saraffova AA, Abouhaidar MG (1997). Unusual effect of clusters of rare arginine (AGG) codons on the expression of human interferon alpha 1 gene in Escherichia coli. Int J Biochem Cell Biol.

[58] Coleman JR, Papamichail D, Skiena S, Futcher B, Wimmer E, Mueller S (2008). Virus attenuation by genome-scale changes in codon pair bias. Science.

[59] de Fabritus L, Nougairède A, Aubry F, Gould EA, de Lamballerie X (2015). Attenuation of tick-borne encephalitis virus using large-scale random codon re-encoding. PLoS Pathog.

[60] Sauna ZE, Kimchi-Sarfaty C (2011). Understanding the contribution of synonymous mutations to human disease. Nat Rev Genet.

[61] Gartner JJ, Parker SC, Prickett TD, Dutton-Regester K, Stitzel ML, Lin JC, Davis S, Simhadri VL, Jha S, Katagiri N (2013). NISC Comparative Sequencing Program: Whole-genome sequencing identifies a recurrent functional synonymous mutation in melanoma. Proc Natl Acad Sci USA.

[62] Ingolia NT (2014). Ribosome profiling: New views of translation, from single codons to genome scale. Nat Rev Genet.

[63] Dana A, Tuller T (2014). The effect of tRNA levels on decoding times of mRNA codons. Nucleic Acids Res.

[64] Fredrick K, Ibba M (2010). How the sequence of a gene can tune its translation. Cell.

[65] Li Q, Qu HQ (2013). Human coding synonymous single nucleotide polymorphisms at ramp regions of mRNA translation. PLoS One.

[66] Charneski CA, Hurst LD (2013). Positively charged residues are the major determinants of ribosomal velocity. PLoS Biol.

[67] Himeno H, Nameki N, Kurita D, Muto A, Abo T (2015). Ribosome rescue systems in bacteria. Biochimie.

[68] Edenberg ER, Downey M, Toczyski D (2014). Polymerase stalling during replication, transcription and translation. Curr Biol.

[69] Faucillion ML, Larsson J (2015). Increased expression of X-linked genes in mammals is associated with a higher stability of transcripts and an increased ribosome density. Genome Biol Evol.

[70] Che F, Fu Q, Li X, Gao N, Qi F, Sun Z, Du Y, Li M (2015). Association of insulin receptor H1085H C>T, insulin receptor substrate 1 G972R and insulin receptor substrate 2 1057G/A polymorphisms with refractory temporal lobe epilepsy in Han Chinese. Seizure.

[71] de la Monte SM, Tong M (2014). Brain metabolic dysfunction at the core of Alzheimer's disease. Biochem Pharmacol.

[72] White MF (2014). IRS2 integrates insulin/IGF1 signalling with metabolism, neurodegeneration and longevity. Diabetes Obes Metab.

[73] de la Monte SM (2012). Contributions of brain insulin resistance and deficiency in amyloid-related neurodegeneration in Alzheimer's disease. Drugs.

[74] Albert-Fort M, Hombrebueno JR, Pons-Vazquez S, Sanz-Gonzalez S, Diaz-Llopis M, Pinazo-Durán MD (2014). Retinal neurodegenerative changes in the adult insulin receptor substrate-2 deficient mouse. Exp Eye Res.

[75] Costello DA, Claret M, Al-Qassab H, Plattner F, Irvine EE, Choudhury AI, Giese KP, Withers DJ, Pedarzani P (2012). Brain deletion of insulin receptor substrate 2 disrupts hippocampal synaptic plasticity and metaplasticity. PLoS One.

[76] Martín ED, Sánchez-Perez A, Trejo JL, Martin-Aldana JA, Cano Jaimez M, Pons S, Acosta Umanzor C, Menes L, White MF, Burks DJ (2012). IRS-2 deficiency impairs NMDA receptor-dependent long-term potentiation. Cereb Cortex.

[77] Sadagurski M, Cheng Z, Rozzo A, Palazzolo I, Kelley GR, Dong X, Krainc D, White MF (2011). IRS2 increases mitochondrial dysfunction and oxidative stress in a mouse model of Huntington disease. J Clin Invest.

[78] Qi Y, Xu Z, Zhu Q, Thomas C, Kumar R, Feng H, Dostal DE, White MF, Baker KM, Guo S (2013). Myocardial loss of IRS1 and IRS2 causes heart failure and is controlled by p38α MAPK during insulin resistance. Diabetes.

[79] Carew RM, Sadagurski M, Goldschmeding R, Martin F, White MF, Brazil DP (2010). Deletion of Irs2 causes reduced kidney size in mice: Role for inhibition of GSK3beta?. BMC Dev Biol.

[80] Hookham MB, O'Donovan HC, Church RH, Mercier-Zuber A, Luzi L, Curran SP, Carew RM, Droguett A, Mezzano S, Schubert M (2013). Insulin receptor substrate-2 is expressed in kidney epithelium and up-regulated in diabetic nephropathy. FEBS J.

[81] Landis J, Shaw LM (2014). Insulin receptor substrate 2-mediated phosphatidylinositol 3-kinase signaling selectively inhibits glycogen synthase kinase 3β to regulate aerobic glycolysis. J Biol Chem.

[82] Porter HA, Perry A, Kingsley C, Tran NL, Keegan AD (2013). IRS1 is highly expressed in localized breast tumors and regulates the sensitivity of breast cancer cells to chemotherapy, while IRS2 is highly expressed in invasive breast tumors. Cancer Lett.

[83] Nishimura R, Takita J, Sato-Otsubo A, Kato M, Koh K, Hanada R, Tanaka Y, Kato K, Maeda D, Fukayama M (2013). Characterization of genetic lesions in rhabdomyosarcoma using a high-density single nucleotide polymorphism array. Cancer Sci.

[84] Verma R, Su S, McCrann DJ, Green JM, Leu K, Young PR, Schatz PJ, Silva JC, Stokes MP, Wojchowski DM (2014). RHEX, a novel regulator of human erythroid progenitor cell expansion and erythroblast development. J Exp Med.

[85] Bunn HF (2013). Erythropoietin. Cold Spring Harb Perspect Med.

[86] Wang H, Rissanen J, Miettinen R, Kärkkäinen P, Kekäläinen P, Kuusisto J, Mykkänen L, Karhapää P, Laakso M (2001). New amino acid substitutions in the IRS-2 gene in Finnish and Chinese subjects with late-onset type 2 diabetes. Diabetes.

[87] Seok J, Warren HS, Cuenca AG, Mindrinos MN, Baker HV, Xu W, Richards DR, McDonald-Smith GP, Gao H, Hennessy L (2013). Inflammation and Host Response to Injury, Large Scale Collaborative Research Program: Genomic responses in mouse models poorly mimic human inflammatory diseases. Proc Natl Acad Sci USA.

[88] Taborsky GJ, Mei Q, Hackney DJ, Mundinger TO (2014). The search for the mechanism of early sympathetic islet neuropathy in autoimmune diabetes. Diabetes Obes Metab.

[89] Nichenametla SN, Lazarus P, Richie JP (2011). A GAG trinucleotide-repeat polymorphism in the gene for glutathione biosynthetic enzyme, GCLC, affects gene expression through translation. FASEB J.

[90] Feuer SK, Liu X, Donjacour A, Lin W, Simbulan RK, Giritharan G, Piane LD, Kolahi K, Ameri K, Maltepe E (2014). Use of a mouse in vitro fertilization model to understand the developmental origins of health and disease hypothesis. Endocrinology.

[91] Campolo J, Penco S, Bianchi E, Colombo L, Parolini M, Caruso R, Sedda V, Patrosso MC, Cighetti G, Marocchi A (2007). Glutamate-cysteine ligase polymorphism, hypertension, and male sex are associated with cardiovascular events. Biochemical and genetic characterization of Italian subpopulation. Am Heart J.

[92] Piao ZH, Kim MS, Jeong M, Yun S, Lee SH, Sun HN, Song HY, Suh HW, Jung H, Yoon SR (2012). VDUP1 exacerbates bacteremic shock in mice infected with Pseudomonas aeruginosa. Cell Immunol.

[93] Shalev A (2014). Minireview: Thioredoxin-interacting protein: regulation and function in the pancreatic β-cell. Mol Endocrinol.

[94] Coucha M, Elshaer SL, Eldahshan WS, Mysona BA, El-Remessy AB (2015). Molecular mechanisms of diabetic retinopathy: Potential therapeutic targets. Middle East Afr J Ophthalmol.

[95] Kaadige MR, Yang J, Wilde BR, Ayer DE (2015). MondoA-Mlx transcriptional activity is limited by mTOR-MondoA interaction. Mol Cell Biol.

[96] Mead EA, Li M, Tu Z, Zhu J (2012). Translational regulation of Anopheles gambiae mRNAs in the midgut during Plasmodium falciparuminfection. BMC Genomics.

[97] Mahajan A, Go MJ, Zhang W, Below JE, Gaulton KJ, Ferreira T, Horikoshi M, Johnson AD, Ng MC, Prokopenko I (2014). DIAbetes Genetics Replication And Meta-analysis (DIAGRAM) Consortium; Asian Genetic Epidemiology Network Type 2 Diabetes (AGEN-T2D) Consortium; South Asian Type 2 Diabetes (SAT2D) Consortium; Mexican American Type 2 Diabetes (MAT2D) Consortium; Type 2 Diabetes Genetic Exploration by Nex-generation sequencing in muylti-Ethnic Samples (T2D-GENES) Consortium: Genome-wide trans-ancestry meta-analysis provides insight into the genetic architecture of type 2 diabetes susceptibility. Nat Genet.

[98] Betarbet R, Anderson LR, Gearing M, Hodges TR, Fritz JJ, Lah JJ, Levey AI (2008). Fas-associated factor 1 and Parkinson's disease. Neurobiol Dis.

[99] Amelio I, Cutruzzolá F, Antonov A, Agostini M, Melino G (2014). Serine and glycine metabolism in cancer. Trends Biochem Sci.

[100] Labaj PP, Leparc GG, Bardet AF, Kreil G, Kreil DP (2010). Single amino acid repeats in signal peptides. FEBS J.

[101] Depledge DP, Dalby AR (2005). COPASAAR - a database for proteomic analysis of single amino acid repeats. BMC Bioinformatics.

[102] Khan A, Hill JM, Adachi M (1970). Inhibition of anti-tumour effect of L-asparaginase by methionine and choline. Lancet.

[103] Rudman D, Vogler WR, Howard CH, Gerron GG (1971). Observations on the plasma amino acids of patients with acute leukemia. Cancer Res.

[104] Jewell JL, Kim YC, Russell RC, Yu FX, Park HW, Plouffe SW, Tagliabracci VS, Guan KL (2015). Metabolism. Differential regulation of mTORC1 by leucine and glutamine. Science.

[105] Jewell JL, Russell RC, Guan KL (2013). Amino acid signalling upstream of mTOR. Nat Rev Mol Cell Biol.

[106] Yang J, Chi Y, Burkhardt BR, Guan Y, Wolf BA (2010). Leucine metabolism in regulation of insulin secretion from pancreatic beta cells. Nutr Rev.

[107] Riedl E, Koeppel H, Brinkkoetter P, Sternik P, Steinbeisser H, Sauerhoefer S, Janssen B, van der Woude FJ, Yard BA (2007). A CTG polymorphism in the CNDP1 gene determines the secretion of serum carnosinase in Cos-7 transfected cells. Diabetes.

[108] Freedman BI, Hicks PJ, Sale MM, Pierson ED, Langefeld CD, Rich SS, Xu J, McDonough C, Janssen B, Yard BA (2007). A leucine repeat in the carnosinase gene CNDP1 is associated with diabetic end-stage renal disease in European Americans. Nephrol Dial Transplant.

[109] Zachariah RM, Olson CO, Ezeonwuka C, Rastegar M (2012). Novel MeCP2 isoform-specific antibody reveals the endogenous MeCP2E1 expression in murine brain, primary neurons and astrocytes. PLoS One.

[110] (1993). The Huntington's Disease Collaborative Research Group: A novel gene containing a trinucleotide repeat that is expanded and unstable on Huntington's disease chromosomes. Cell.

[111] Klesert TR, Otten AD, Bird TD, Tapscott SJ (1997). Trinucleotide repeat expansion at the myotonic dystrophy locus reduces expression of DMAHP. Nat Genet.

[112] Korade-Mirnics Z, Babitzke P, Hoffman E (1998). Myotonic dystrophy: Molecular windows on a complex etiology. Nucleic Acids Res.

[113] Lozano R, Rosero CA, Hagerman RJ (2014). Fragile X spectrum disorders. Intractable Rare Dis Res.

[114] Laaksovirta H, Peuralinna T, Schymick JC, Scholz SW, Lai SL, Myllykangas L, Sulkava R, Jansson L, Hernandez DG, Gibbs JR (2010). Chromosome 9p21 in amyotrophic lateral sclerosis in Finland: A genome-wide association study. Lancet Neurol.

[115] Gijselinck I, Van Langenhove T, van der Zee J, Sleegers K, Philtjens S, Kleinberger G, Janssens J, Bettens K, Van Cauwenberghe C, Pereson S (2012). A C9orf72 promoter repeat expansion in a Flanders-Belgian cohort with disorders of the frontotemporal lobar degeneration-amyotrophic lateral sclerosis spectrum: A gene identification study. Lancet Neurol.

[116] Rohrer JD, Isaacs AM, Mizielinska S, Mead S, Lashley T, Wray S, Sidle K, Fratta P, Orrell RW, Hardy J (2015). C9orf72 expansions in frontotemporal dementia and amyotrophic lateral sclerosis. Lancet Neurol.

[117] Walsh MJ, Cooper-Knock J, Dodd JE, Stopford MJ, Mihaylov SR, Kirby J, Shaw PJ, Hautbergue GM (2015). Invited review: decoding the pathophysiological mechanisms that underlie RNA dysregulation in neurodegenerative disorders: a review of the current state of the art. Neuropathol Appl Neurobiol.

[118] Cleary JD, Ranum LP (2014). Repeat associated non-ATG (RAN) translation: New starts in microsatellite expansion disorders. Curr Opin Genet Dev.

[119] Yan S, Wen JD, Bustamante C, Tinoco I (2015). Ribosome excursions during mRNA translocation mediate broad branching of frameshift pathways. Cell.

[120] Scoles DR, Ho MH, Dansithong W, Pflieger LT, Petersen LW, Thai KK, Pulst SM (2015). Repeat Associated Non-AUG Translation (RAN Translation) Dependent on Sequence Downstream of the ATXN2 CAG Repeat. PLoS One.

[121] Muerdter F, Stark A (2014). Genomics: Hiding in plain sight. Nature.

[122] La Spada AR, Paulson HL, Fischbeck KH (1994). Trinucleotide repeat expansion in neurological disease. Ann Neurol.

[123] Kayatekin C, Matlack KE, Hesse WR, Guan Y, Chakrabortee S, Russ J, Wanker EE, Shah JV, Lindquist S (2014). Prion-like proteins sequester and suppress the toxicity of huntingtin exon 1. Proc Natl Acad Sci USA.

[124] Ripaud L, Chumakova V, Antonin M, Hastie AR, Pinkert S, Körner R, Ruff KM, Pappu RV, Hornburg D, Mann M (2014). Overexpression of Q-rich prion-like proteins suppresses polyQ cytotoxicity and alters the polyQ interactome. Proc Natl Acad Sci USA.

[125] Chambers JW, Maguire TG, Alwine JC (2010). Glutamine metabolism is essential for human cytomegalovirus infection. J Virol.

[126] Mangiarini L, Sathasivam K, Seller M, Cozens B, Harper A, Hetherington C, Lawton M, Trottier Y, Lehrach H, Davies SW (1996). Exon 1 of the HD gene with an expanded CAG repeat is sufficient to cause a progressive neurological phenotype in transgenic mice. Cell.

[127] Rosas HD, Reuter M, Doros G, Lee SY, Triggs T, Malarick K, Fischl B, Salat DH, Hersch SM (2011). A tale of two factors: what determines the rate of progression in Huntington's disease? A longitudinal MRI study. Mov Disord.

[128] Lee JM, Ramos EM, Lee JH, Gillis T, Mysore JS, Hayden MR, Warby SC, Morrison P, Nance M, Ross CA (2012). PREDICT-HD study of the Huntington Study Group (HSG); REGISTRY study of the European Huntington's Disease Network; HD-MAPS Study Group; COHORT study of the HSG: CAG repeat expansion in Huntington disease determines age at onset in a fully dominant fashion. Neurology.

[129] Qin J, Li Y, Cai Z, Li S, Zhu J, Zhang F, Liang S, Zhang W, Guan Y, Shen D (2012). A metagenome-wide association study of gut microbiota in type 2 diabetes. Nature.

[130] Ohlsson C, Sjögren K (2015). Effects of the gut microbiota on bone mass. Trends Endocrinol Metab.

[131] DelGiorno KE, Tam JW, Hall JC, Thotakura G, Crawford HC, van der Velden AW (2014). Persistent salmonellosis causes pancreatitis in a murine model of infection. PLoS One.

[132] Whitcomb DC (2010). Genetic aspects of pancreatitis. Annu Rev Med.

[133] Wu F, Qu L, Tan Y, Zhang Y, Hu C (2014). L-asparaginase-induced severe acute pancreatitis in an adult with extranodal natural killer/T-cell lymphoma, nasal type: A case report and review of the literature. Oncol Lett.

[134] Kaya I, Citil M, Sozmen M, Karapehlivan M, Cigsar G (2014). Investigation of protective effect of L-carnitine on L-asparaginase-induced acute pancreatic injury in male Balb/c mice. Dig Dis Sci.

[135] Bueno SM, Riquelme S, Riedel CA, Kalergis AM (2012). Mechanisms used by virulent Salmonella to impair dendritic cell function and evade adaptive immunity. Immunology.

[136] Kafkewitz D, Bendich A (1983). Enzyme-induced asparagine and glutamine depletion and immune system function. Am J Clin Nutr.

[137] Etheredge EE, Shons A, Harris N, Najarian JS (1971). Prolongation of skin xenograft survival by L-asparaginase. Transplantation.

[138] Khan A, Levine S (1974). Further studies on the inhibition of allergic encephalomyelitis by L-asparaginase. J Immunol.

[139] Friedman H (1971). L-asparaginase induced immunosuppression: Inhibition of bone marrow derived antibody precursor cells. Science.

[140] Xu J, Wang P, Li Y, Li G, Kaczmarek LK, Wu Y, Koni PA, Flavell RA, Desir GV (2004). The voltage-gated potassium channel Kv1.3 regulates peripheral insulin sensitivity. Proc Natl Acad Sci USA.

[141] Wang T, Lee MH, Choi E, Pardo-Villamizar CA, Lee SB, Yang IH, Calabresi PA, Nath A (2012). Granzyme B-induced neurotoxicity is mediated via activation of PAR-1 receptor and Kv1.3 channel. PLoS One.

[142] LaRusch J, Whitcomb DC (2011). Genetics of pancreatitis. Curr Opin Gastroenterol.

[143] Blackman SM, Commander CW, Watson C, Arcara KM, Strug LJ, Stonebraker JR, Wright FA, Rommens JM, Sun L, Pace RG (2013). Genetic modifiers of cystic fibrosis-related diabetes. Diabetes.

[144] Santoro N, Colombini A, Silvestri D, Grassi M, Giordano P, Parasole R, Barisone E, Caruso R, Conter V, Valsecchi MG (2013). Screening for coagulopathy and identification of children with acute lymphoblastic leukemia at a higher risk of symptomatic venous thrombosis: An AIEOP experience. J Pediatr Hematol Oncol.

[145] Ellinghaus E, Stanulla M, Richter G, Ellinghaus D, te Kronnie G, Cario G, Cazzaniga G, Horstmann M, Panzer Grümayer R, Cavé H (2012). Identification of germline susceptibility loci in ETV6-RUNX1-rearranged childhood acute lymphoblastic leukemia. Leukemia.

[146] Xu J, Koni PA, Wang P, Li G, Kaczmarek L, Wu Y, Li Y, Flavell RA, Desir GV (2003). The voltage-gated potassium channel Kv1.3 regulates energy homeostasis and body weight. Hum Mol Genet.

[147] Tu L, Khanna P, Deutsch C (2014). Transmembrane segments form tertiary hairpins in the folding vestibule of the ribosome. J Mol Biol.

[148] Kosolapov A, Deutsch C (2009). Tertiary interactions within the ribosomal exit tunnel. Nat Struct Mol Biol.

[149] Delaney E, Khanna P, Tu L, Robinson JM, Deutsch C (2014). Determinants of pore folding in potassium channel biogenesis. Proc Natl Acad Sci USA.

[150] Ko SB, Zeng W, Dorwart MR, Luo X, Kim KH, Millen L, Goto H, Naruse S, Soyombo A, Thomas PJ (2004). Gating of CFTR by the STAS domain of SLC26 transporters. Nat Cell Biol.

[151] Gray MA (2004). Bicarbonate secretion: It takes two to tango. Nat Cell Biol.

[152] Chang MH, Plata C, Sindic A, Ranatunga WK, Chen AP, Zandi-Nejad K, Chan KW, Thompson J, Mount DB, Romero MF (2009). Slc26a9 is inhibited by the R-region of the cystic fibrosis transmembrane conductance regulator via the STAS domain. J Biol Chem.

[153] Ishiguro H, Yamamoto A, Nakakuki M, Yi L, Ishiguro M, Yamaguchi M, Kondo S, Mochimaru Y (2012). Physiology and pathophysiology of bicarbonate secretion by pancreatic duct epithelium. Nagoya J Med Sci.

[154] Kimchi-Sarfaty C, Oh JM, Kim IW, Sauna ZE, Calcagno AM, Ambudkar SV, Gottesman MM (2007). A 'silent' polymorphism in the MDR1 gene changes substrate specificity. Science.

[155] Chong PA, Kota P, Dokholyan NV, Forman-Kay JD (2013). Dynamics intrinsic to cystic fibrosis transmembrane conductance regulator function and stability. Cold Spring Harb Perspect Med.

[156] LaRusch J, Jung J, General IJ, Lewis MD, Park HW, Brand RE, Gelrud A, Anderson MA, Banks PA, Conwell D, North American Pancreatitis Study Group (2014). Mechanisms of CFTR functional variants that impair regulated bicarbonate permeation and increase risk for pancreatitis but not for cystic fibrosis. PLoS Genet.

[157] El Khouri E, Touré A (2014). Functional interaction of the cystic fibrosis transmembrane conductance regulator with members of the SLC26 family of anion transporters (SLC26A8 and SLC26A9): Physiological and pathophysiological relevance. Int J Biochem Cell Biol.

[158] Bozoky Z, Krzeminski M, Muhandiram R, Birtley JR, Al-Zahrani A, Thomas PJ, Frizzell RA, Ford RC, Forman-Kay JD (2013). Regulatory R region of the CFTR chloride channel is a dynamic integrator of phospho-dependent intra-and intermolecular interactions. Proc Natl Acad Sci USA.

[159] Pier GB, Grout M, Zaidi T, Meluleni G, Mueschenborn SS, Banting G, Ratcliff R, Evans MJ, Colledge WH (1998). Salmonella typhi uses CFTR to enter intestinal epithelial cells. Nature.

[160] Lazrak A, Fu L, Bali V, Bartoszewski R, Rab A, Havasi V, Keiles S, Kappes J, Kumar R, Lefkowitz E (2013). The silent codon change I507-ATC->ATT contributes to the severity of the DeltaF508 CFTR channel dysfunction. FASEB J.

[161] van der Wijst J, Bindels RJ, Hoenderop JG (2014). Mg^2+^ homeostasis: The balancing act of TRPM6. Curr Opin Nephrol Hypertens.

[162] Smith JG, Avery CL, Evans DS, Nalls MA, Meng YA, Smith EN, Palmer C, Tanaka T, Mehra R, Butler AM (2012). CARe and COGENT consortia: Impact of ancestry and common genetic variants on QT interval in African Americans. Circ Cardiovasc Genet.

[163] Hermosura MC, Garruto RM (2007). TRPM7 and TRPM2-Candidate susceptibility genes for Western Pacific ALS and PD?. Biochim Biophys Acta.

[164] Krapivinsky G, Krapivinsky L, Manasian Y, Clapham DE (2014). The TRPM7 chanzyme is cleaved to release a chromatin-modifying kinase. Cell.

[165] Wrighton KH (2014). Epigenetics: The TRPM7 ion channel modifies histones. Nat Rev Mol Cell Biol.

[166] Zeng Z, Inoue K, Sun H, Leng T, Feng X, Zhu L, Xiong ZG (2015). TRPM7 regulates vascular endothelial cell adhesion and tube formation. Am J Physiol Cell Physiol.

[167] Chen JP, Wang J, Luan Y, Wang CX, Li WH, Zhang JB, Sha D, Shen R, Cui YG, Zhang Z (2015). TRPM7 promotes the metastatic process in human nasopharyngeal carcinoma. Cancer Lett.

[168] Hunt RC, Simhadri VL, Iandoli M, Sauna ZE, Kimchi-Sarfaty C (2014). Exposing synonymous mutations. Trends Genet.

[169] Wu DF, Yin RX, Cao XL, Chen WX, Aung LH, Wang W, Huang KK, Huang P, Zeng XN, Wu J (2013). Scavenger receptor class B type 1 gene rs5888 single nucleotide polymorphism and the risk of coronary artery disease and ischemic stroke: A case-control study. Int J Med Sci.

[170] Constantineau J, Greason E, West M, Filbin M, Kieft JS, Carletti MZ, Christenson LK, Rodriguez A (2010). A synonymous variant in scavenger receptor, class B, type I gene is associated with lower SR-BI protein expression and function. Atherosclerosis.

[171] Willer CJ, Schmidt EM, Sengupta S, Peloso GM, Gustafsson S, Kanoni S, Ganna A, Chen J, Buchkovich ML, Mora S (2013). Global Lipids Genetics Consortium: Discovery and refinement of loci associated with lipid levels. Nat Genet.

[172] Meyer JM, Graf GA, van der Westhuyzen DR (2013). New developments in selective cholesteryl ester uptake. Curr Opin Lipidol.

[173] Nofer JR (2015). Signal transduction by HDL: Agonists, receptors, and signaling cascades. Handb Exp Pharmacol.

[174] Tong WH, Pieters R, de Groot-Kruseman HA, Hop WC, Boos J, Tissing WJ, van der Sluis IM (2014). The toxicity of very prolonged courses of PEGasparaginase or Erwinia asparaginase in relation to asparaginase activity, with a special focus on dyslipidemia. Haematologica.

[175] Stanislovaitiene D, Lesauskaite V, Zaliuniene D, Smalinskiene A, Gustiene O, Zaliaduonyte-Peksiene D, Tamosiunas A, Luksiene D, Petkeviciene J, Zaliunas R (2013). SCARB1 single nucleotide polymorphism (rs5888) is associated with serum lipid profile and myocardial infarction in an age- and gender-dependent manner. Lipids Health Dis.

[176] Purdue MP, Johansson M, Zelenika D, Toro JR, Scelo G, Moore LE, Prokhortchouk E, Wu X, Kiemeney LA, Gaborieau V (2011). Genome-wide association study of renal cell carcinoma identifies two susceptibility loci on 2p21 and 11q13.3. Nat Genet.

[177] Pośpiech E, Ligęza J, Wilk W, Gołas A, Jaszczyński J, Stelmach A, Ryś J, Blecharczyk A, Wojas-Pelc A, Jura J (2015). Variants of SCARB1 and VDR involved in complex genetic interactions may be implicated in the genetic susceptibility to clear cell renal cell carcinoma. Biomed Res Int.

[178] Suchindran S, Rivedal D, Guyton JR, Milledge T, Gao X, Benjamin A, Rowell J, Ginsburg GS, McCarthy JJ (2010). Genome-wide association study of Lp-PLA(2) activity and mass in the Framingham Heart Study. PLoS Genet.

[179] Song GJ, Kim SM, Park KH, Kim J, Choi I, Cho KH (2015). SR-BI mediates high density lipoprotein (HDL)-induced anti-inflammatory effect in macrophages. Biochem Biophys Res Commun.

[180] Gao M, Zhao D, Schouteden S, Sorci-Thomas MG, Van Veldhoven PP, Eggermont K, Liu G, Verfaillie CM, Feng Y (2014). Regulation of high-density lipoprotein on hematopoietic stem/progenitor cells in atherosclerosis requires scavenger receptor type BI expression. Arterioscler Thromb Vasc Biol.

[181] Sticozzi C, Belmonte G, Cervellati F, Muresan XM, Pessina F, Lim Y, Forman HJ, Valacchi G (2014). Resveratrol protects SR-B1 levels in keratinocytes exposed to cigarette smoke. Free Radic Biol Med.

[182] Christianson MS, Yates M (2012). Scavenger receptor class B type 1 gene polymorphisms and female fertility. Curr Opin Endocrinol Diabetes Obes.

[183] Meyers KJ, Mares JA, Igo RP, Truitt B, Liu Z, Millen AE, Klein M, Johnson EJ, Engelman CD, Karki CK (2014). Genetic evidence for role of carotenoids in age-related macular degeneration in the carotenoids in age-related eye disease study (CAREDS). Invest Ophthalmol Vis Sci.

[184] Reboul E, Goncalves A, Comera C, Bott R, Nowicki M, Landrier JF, Jourdheuil-Rahmani D, Dufour C, Collet X, Borel P (2011). Vitamin D intestinal absorption is not a simple passive diffusion: Evidences for involvement of cholesterol transporters. Mol Nutr Food Res.

[185] Goncalves A, Margier M, Roi S, Collet X, Niot I, Goupy P, Caris-Veyrat C, Reboul E (2014). Intestinal scavenger receptors are involved in vitamin K1 absorption. J Biol Chem.

[186] Major JM, Yu K, Wheeler W, Zhang H, Cornelis MC, Wright ME, Yeager M, Snyder K, Weinstein SJ, Mondul A (2011). Genome-wide association study identifies common variants associated with circulating vitamin E levels. Hum Mol Genet.

[187] Schulman S, Furie B (2015). How I treat poisoning with vitamin K antagonists. Blood.

[188] Ibarrola-Jurado N, Salas-Salvadó J, Martínez-González MA, Bulló M (2012). Dietary phylloquinone intake and risk of type 2 diabetes in elderly subjects at high risk of cardiovascular disease. Am J Clin Nutr.

[189] Tang W, Schwienbacher C, Lopez LM, Ben-Shlomo Y, Oudot-Mellakh T, Johnson AD, Samani NJ, Basu S, Gögele M, Davies G (2012). Genetic associations for activated partial thromboplastin time and prothrombin time, their gene expression profiles, and risk of coronary artery disease. Am J Hum Genet.

[190] Melville SA, Buros J, Parrado AR, Vardarajan B, Logue MW, Shen L, Risacher SL, Kim S, Jun G, DeCarli C (2012). Alzheimer's Disease Neuroimaging Initiative: Multiple loci influencing hippocampal degeneration identified by genome scan. Ann Neurol.

[191] Nowak-Göttl U, Wermes C, Junker R, Koch HG, Schobess R, Fleischhack G, Schwabe D, Ehrenforth S (1999). Prospective evaluation of the thrombotic risk in children with acute lymphoblastic leukemia carrying the MTHFR TT 677 genotype, the prothrombin G20210A variant, and further prothrombotic risk factors. Blood.

[192] Schmalbach B, Stepanow O, Jochens A, Riedel C, Deuschl G, Kuhlenbäumer G (2015). Determinants of platelet-leukocyte aggregation and platelet activation in stroke. Cerebrovasc Dis.

[193] Gieger C, Radhakrishnan A, Cvejic A, Tang W, Porcu E, Pistis G, Serbanovic-Canic J, Elling U, Goodall AH, Labrune Y (2011). New gene functions in megakaryopoiesis and platelet formation. Nature.

[194] Hunault-Berger M, Chevallier P, Delain M, Bulabois CE, Bologna S, Bernard M, Lafon I, Cornillon J, Maakaroun A, Tizon A, GOELAMS (Groupe Ouest-Est des Leucémies Aiguës et Maladies du Sang) (2008). Changes in antithrombin and fibrinogen levels during induction chemotherapy with L-asparaginase in adult patients with acute lymphoblastic leukemia or lymphoblastic lymphoma. Use of supportive coagulation therapy and clinical outcome: The CAPELAL study Haematologica.

[195] López Herce Cid J, Martínez A, González M, García S (1986). Diabetic ketoacidosis and hypofibrinogenemia as a complication of the treatment with L-asparaginase of acute lymphoblastic leukemia. Sangre (Barc).

[196] Alving BM, Barr CF, Tang DB (1984). L-asparaginase: Acute effects on protein synthesis in rabbits with normal and increased fibrinogen production. Blood.

[197] Brodsky I, Kahn SB, Vash G, Ross EM, Petkov G (1971). Fibrinogen survival with [75Se]Selenomethionine during L-asparaginase therapy. Br J Haematol.

[198] Sleddering MA, Markvoort AJ, Dharuri HK, Jeyakar S, Snel M, Juhasz P, Lynch M, Hines W, Li X, Jazet IM (2014). Proteomic analysis in type 2 diabetes patients before and after a very low calorie diet reveals potential disease state and intervention specific biomarkers. PLoS One.

[199] Barazzoni R, Kiwanuka E, Zanetti M, Cristini M, Vettore M, Tessari P (2003). Insulin acutely increases fibrinogen production in individuals with type 2 diabetes but not in individuals without diabetes. Diabetes.

[200] Luo C, Zhao J, Madden A, Chen M, Xu H (2013). Complement expression in retinal pigment epithelial cells is modulated by activated macrophages. Exp Eye Res.

[201] Kallio SP, Jakkula E, Purcell S, Suvela M, Koivisto K, Tienari PJ, Elovaara I, Pirttilä T, Reunanen M, Bronnikov D (2009). Use of a genetic isolate to identify rare disease variants: C7 on 5p associated with MS. Hum Mol Genet.

[202] Brudner M, Karpel M, Lear C, Chen L, Yantosca LM, Scully C, Sarraju A, Sokolovska A, Zariffard MR, Eisen DP (2013). Lectin-dependent enhancement of Ebola virus infection via soluble and transmembrane C-type lectin receptors. PLoS One.

[203] van Vliet SJ, Steeghs L, Bruijns SC, Vaezirad MM, Snijders Blok C, Arenas Busto JA, Deken M, van Putten JP, van Kooyk Y (2009). Variation of Neisseria gonorrhoeae lipooligosaccharide directs dendritic cell-induced T helper responses. PLoS Pathog.

[204] Chen P, Zhang Q, Dang H, Liu X, Tian F, Zhao J, Chen Y, Zhang H, Chen W (2014). Antidiabetic effect of Lactobacillus casei CCFM0412 on mice with type 2 diabetes induced by a high-fat diet and streptozotocin. Nutrition.

[205] Meyre D, Pare G (2013). Genetic dissection of diabetes: Facing the giant. Diabetes.

[206] Qi L, Parast L, Cai T, Powers C, Gervino EV, Hauser TH, Hu FB, Doria A (2011). Genetic susceptibility to coronary heart disease in type 2 diabetes: 3 independent studies. J Am Coll Cardiol.

[207] Sandyk R (1993). The relationship between diabetes mellitus and Parkinson's disease. Int J Neurosci.

[208] Calkin CV, Ruzickova M, Uher R, Hajek T, Slaney CM, Garnham JS, O'Donovan MC, Alda M (2015). Insulin resistance and outcome in bipolar disorder. Br J Psychiatry.

[209] Cosgrove J, Alty JE, Jamieson S (2015). Cognitive impairment in Parkinson's disease. Postgrad Med J.

[210] Talbot K (2014). Amyotrophic lateral sclerosis: cell vulnerability or system vulnerability?. J Anat.

[211] Carbutt S, Duff J, Yarnall A, Burn DJ, Hudson G (2015). Variation in complement protein C1q is not a major contributor to cognitive impairment in Parkinson's disease. Neurosci Lett.

[212] Ressl S, Vu BK, Vivona S, Martinelli DC, Südhof TC, Brunger AT (2015). Structures of C1q-like proteins reveal unique features among the C1q/TNF superfamily. Structure.

[213] Sigoillot SM, Iyer K, Binda F, González-Calvo I, Talleur M, Vodjdani G, Isope P, Selimi F (2015). The secreted protein C1QL1 and its receptor BAI3 control the synaptic connectivity of excitatory inputs converging on cerebellar purkinje cells. Cell Rep.

[214] Wang K, Zhang H, Ma D, Bucan M, Glessner JT, Abrahams BS, Salyakina D, Imielinski M, Bradfield JP, Sleiman PM (2009). Common genetic variants on 5p14.1 associate with autism spectrum disorders. Nature.

[215] Malenfant P, Liu X, Hudson ML, Qiao Y, Hrynchak M, Riendeau N, Hildebrand MJ, Cohen IL, Chudley AE, Forster-Gibson C (2012). Association of GTF2i in the Williams-Beuren syndrome critical region with autism spectrum disorders. J Autism Dev Disord.

[216] Lu RC, Wang H, Tan MS, Yu JT, Tan L (2014). TMEM106B and APOE polymorphisms interact to confer risk for late-onset Alzheimer's disease in Han Chinese. J Neural Transm.

[217] Stagi M, Klein ZA, Gould TJ, Bewersdorf J, Strittmatter SM (2014). Lysosome size, motility and stress response regulated by fronto-temporal dementia modifier TMEM106B. Mol Cell Neurosci.

[218] Paoletti C, Hayes DF (2014). Molecular testing in breast cancer. Annu Rev Med.

[219] Ma CX, Reinert T, Chmielewska I, Ellis MJ (2015). Mechanisms of aromatase inhibitor resistance. Nat Rev Cancer.

[220] Nollau P, Wolters-Eisfeld G, Mortezai N, Kurze AK, Klampe B, Debus A, Bockhorn M, Niendorf A, Wagener C (2013). Protein domain histochemistry (PDH): binding of the carbohydrate recognition domain (CRD) of recombinant human glycoreceptor CLEC10A (CD301) to formalin-fixed, paraffin-embedded breast cancer tissues. J Histochem Cytochem.

[221] Chen W, Salto-Tellez M, Palanisamy N, Ganesan K, Hou Q, Tan LK, Sii LH, Ito K, Tan B, Wu J (2007). Targets of genome copy number reduction in primary breast cancers identified by integrative genomics. Genes Chromosomes Cancer.

[222] Ahmeti KB, Ajroud-Driss S, Al-Chalabi A, Andersen PM, Armstrong J, Birve A, Blauw HM, Brown RH, Bruijn L, Chen W (2013). Age of onset of amyotrophic lateral sclerosis is modulated by a locus on 1p34.1. Neurobiol Aging.

[223] Brady OA, Zheng Y, Murphy K, Huang M, Hu F (2013). The frontotemporal lobar degeneration risk factor, TMEM106B, regulates lysosomal morphology and function. Hum Mol Genet.

[224] Sergouniotis PI, Chakarova C, Murphy C, Becker M, Lenassi E, Arno G, Lek M, MacArthur DG, Bhattacharya SS, Moore AT (2014). UCL-Exomes Consortium: Biallelic variants in TTLL5, encoding a tubulin glutamylase, cause retinal dystrophy. Am J Hum Genet.

[225] Dichgans M, Malik R, König IR, Rosand J, Clarke R, Gretarsdottir S, Thorleifsson G, Mitchell BD, Assimes TL, Levi C (2014). METASTROKE Consortium; CARDIoGRAM Consortium; C4D Consortium; International Stroke Genetics Consortium: Shared genetic susceptibility to ischemic stroke and coronary artery disease: A genome-wide analysis of common variants. Stroke.

[226] Hartmaier RJ, Richter AS, Gillihan RM, Sallit JZ, McGuire SE, Wang J, Lee AV, Osborne CK, O'Malley BW, Brown PH (2012). A SNP in steroid receptor coactivator-1 disrupts a GSK3β phosphorylation site and is associated with altered tamoxifen response in bone. Mol Endocrinol.

[227] Pharoah PD, Tsai YY, Ramus SJ, Phelan CM, Goode EL, Lawrenson K, Buckley M, Fridley BL, Tyrer JP, Shen H (2013). GWAS meta-analysis and replication identifies three new susceptibility loci for ovarian cancer. Nat Genet.

[228] Kriegel MA, Rathinam C, Flavell RA (2009). E3 ubiquitin ligase GRAIL controls primary T cell activation and oral tolerance. Proc Natl Acad Sci USA.

[229] MacKenzie DA, Schartner J, Lin J, Timmel A, Jennens-Clough M, Fathman CG, Seroogy CM (2007). GRAIL is up-regulated in CD4^+^ CD25^+^ T regulatory cells and is sufficient for conversion of T cells to a regulatory phenotype. J Biol Chem.

[230] Seroogy CM1, Soares L, Ranheim EA, Su L, Holness C, Bloom D, Fathman CG (2004). The gene related to anergy in lymphocytes, an E3 ubiquitin ligase, is necessary for anergy induction in CD4 T cells. J Immunol.

[231] (1895). A death attributed to antitoxin. Boston Med Surg J.

[232] Hunt EL (1943). Death from allergic shock. N Engl J Med.

[233] Kortright JL (1896). Practical experiences with antitoxin. Brooklyn MJ (Medical Society of the County of Kings).

[234] Gillis C, Gouel-Chéron A, Jönsson F, Bruhns P (2014). Contribution of human FcγRs to disease with evidence from human polymorphisms and transgenic animal studies. Front Immunol.

[235] Lu W, Lin C, Li Y (2014). Rottlerin induces Wnt co-receptor LRP6 degradation and suppresses both Wnt/β-catenin and mTORC1 signaling in prostate and breast cancer cells. Cell Signal.

[236] Malinauskas T, Jones EY (2014). Extracellular modulators of Wnt signalling. Curr Opin Struct Biol.

[237] Joiner DM, Ke J, Zhong Z, Xu HE, Williams BO (2013). LRP5 and LRP6 in development and disease. Trends Endocrinol Metab.

[238] Moon RT, Kohn AD, De Ferrari GV, Kaykas A (2004). WNT and beta-catenin signalling: Diseases and therapies. Nat Rev Genet.

[239] Jiang X, Charlat O, Zamponi R, Yang Y, Cong F (2015). Dishevelled Promotes Wnt Receptor Degradation through Recruitment of ZNRF3/RNF43 E3 Ubiquitin Ligases. Mol Cell.

[240] Holland J, Fasanello S, Onuma T (1974). Psychiatric symptoms associated with L-asparaginase administration. J Psychiatr Res.

[241] Feinberg WM, Swenson MR (1988). Cerebrovascular complications of L-asparaginase therapy. Neurology.

[242] Rodrigo R, Cauli O, Boix J, ElMlili N, Agusti A, Felipo V (2009). Role of NMDA receptors in acute liver failure and ammonia toxicity: Therapeutical implications. Neurochem Int.

[243] Davidovic L, Jaglin XH, Lepagnol-Bestel AM, Tremblay S, Simonneau M, Bardoni B, Khandjian EW (2007). The fragile X mental retardation protein is a molecular adaptor between the neurospecific KIF3C kinesin and dendritic RNA granules. Hum Mol Genet.

[244] Darnell JC, Klann E (2013). The translation of translational control by FMRP: Therapeutic targets for FXS. Nat Neurosci.

[245] Poliakov E, Koonin EV, Rogozin IB (2014). Impairment of translation in neurons as a putative causative factor for autism. Biol Direct.

[246] Cauchi RJ (2014). Gem depletion: Amyotrophic lateral sclerosis and spinal muscular atrophy crossover. CNS Neurosci Ther.

[247] Häggmark A, Mikus M, Mohsenchian A, Hong MG, Forsström B, Gajewska B, Barańczyk-Kuźma A, Uhlén M, Schwenk JM, Kuźma-Kozakiewicz M (2014). Plasma profiling reveals three proteins associated to amyotrophic lateral sclerosis. Ann Clin Transl Neurol.

[248] Ingre C, Roos PM, Piehl F, Kamel F, Fang F (2015). Risk factors for amyotrophic lateral sclerosis. Clin Epidemiol.

[249] Smith WW, Liu Z, Liang Y, Masuda N, Swing DA, Jenkins NA, Copeland NG, Troncoso JC, Pletnikov M, Dawson TM (2010). Synphilin-1 attenuates neuronal degeneration in the A53T alpha-synuclein transgenic mouse model. Hum Mol Genet.

[250] Wang X, Zeng W, Kim MS, Allen PB, Greengard P, Muallem S (2007). Spinophilin/neurabin reciprocally regulate signaling intensity by G protein-coupled receptors. EMBO J.

[251] Latourelle JC, Pankratz N, Dumitriu A, Wilk JB, Goldwurm S, Pezzoli G, Mariani CB, DeStefano AL, Halter C, Gusella JF (2009). PROGENI Investigators, Coordinators and Molecular Genetic Laboratories; GenePD Investigators, Coordinators and Molecular Genetic Laboratories: Genomewide association study for onset age in Parkinson disease. BMC Med Genet.

[252] Lalla E, Papapanou PN (2011). Diabetes mellitus and periodontitis: A tale of two common interrelated diseases. Nat Rev Endocrinol.

[253] Zeng Z, Feingold E, Wang X, Weeks DE, Lee M, Cuenco DT, Broffitt B, Weyant RJ, Crout R, McNeil DW (2014). Genome-wide association study of primary dentition pit-and-fissure and smooth surface caries. Caries Res.

[254] Teumer A, Holtfreter B, Völker U, Petersmann A, Nauck M, Biffar R, Völzke H, Kroemer HK, Meisel P, Homuth G (2013). Genome-wide association study of chronic periodontitis in a general German population. J Clin Periodontol.

[255] Elks CE, Perry JR, Sulem P, Chasman DI, Franceschini N, He C, Lunetta KL, Visser JA, Byrne EM, Cousminer DL (2010). GIANT Consortium: Thirty new loci for age at menarche identified by a meta-analysis of genome-wide association studies. Nat Genet.

[256] Haas J, Beer AG, Widschwendter P, Oberdanner J, Salzmann K, Sarg B, Lindner H, Herz J, Patsch JR, Marschang P (2011). LRP1b shows restricted expression in human tissues and binds to several extracellular ligands, including fibrinogen and apoE-carrying lipoproteins. Atherosclerosis.

[257] Poduslo SE, Huang R, Spiro A (2010). A genome screen of successful aging without cognitive decline identifies LRP1B by haplotype analysis. Am J Med Genet B Neuropsychiatr Genet.

[258] Scheffer DI, Zhang DS, Shen J, Indzhykulian A, Karavitaki KD, Xu YJ, Wang Q, Lin JJ, Chen ZY, Corey DP (2015). XIRP2, an Actin-Binding Protein Essential for Inner Ear Hair-Cell Stereocilia. Cell Rep.

[259] Francis SP, Krey JF, Krystofiak ES, Cui R, Nanda S, Xu W, Kachar B, Barr-Gillespie PG, Shin JB (2015). A short splice form of Xin-actin binding repeat containing 2 (XIRP2) lacking the Xin repeats is required for maintenance of stereocilia morphology and hearing function. J Neurosci.

[260] Nielsen DA, Ji F, Yuferov V, Ho A, He C, Ott J, Kreek MJ (2010). Genome-wide association study identifies genes that may contribute to risk for developing heroin addiction. Psychiatr Genet.

[261] McCalmon SA, Desjardins DM, Ahmad S, Davidoff KS, Snyder CM, Sato K, Ohashi K, Kielbasa OM, Mathew M, Ewen EP (2010). Modulation of angiotensin II-mediated cardiac remodeling by the MEF2A target gene Xirp2. Circ Res.

[262] Wang Q, Lin JL, Erives AJ, Lin CI, Lin JJ (2014). New insights into the roles of Xin repeat-containing proteins in cardiac development, function, and disease. Int Rev Cell Mol Biol.

[263] Matsuoka R, Abe S, Tokoro F, Arai M, Noda T, Watanabe S, Horibe H, Fujimaki T, Oguri M, Kato K (2015). Association of six genetic variants with myocardial infarction. Int J Mol Med.

[264] Roy A, Guatimosim S, Prado VF, Gros R, Prado MA (2014). Cholinergic activity as a new target in diseases of the heart. Mol Med.

[265] Zhang J, Fan J, Venneti S, Cross JR, Takagi T, Bhinder B, Djaballah H, Kanai M, Cheng EH, Judkins AR (2014). Asparagine plays a critical role in regulating cellular adaptation to glutamine depletion. Mol Cell.

[266] Treviño LR, Yang W, French D, Hunger SP, Carroll WL, Devidas M, Willman C, Neale G, Downing J, Raimondi SC (2009). Germline genomic variants associated with childhood acute lymphoblastic leukemia. Nat Genet.

[267] Seghatoleslam A, Monabati A, Bozorg-Ghalati F, Nikseresht M, Bordbar MR, Rahvar M, Owji AA (2012). Expression of UBE2Q2, a putative member of the ubiquitin-conjugating enzyme family in pediatric acute lymphoblastic leukemia. Arch Iran Med.

[268] Velma V, Broome HJ, Hebert MD (2012). Regulated specific proteolysis of the Cajal body marker protein coilin. Chromosoma.

[269] Gubanova E, Brown B, Ivanov SV, Helleday T, Mills GB, Yarbrough WG, Issaeva N (2012). Downregulation of SMG-1 in HPV-positive head and neck squamous cell carcinoma due to promoter hypermethylation correlates with improved survival. Clin Cancer Res.

[270] Diamond G, Cedar H, Marcus M (1989). A temperature-sensitive mutation in asparaginyl-tRNA synthetase causes cell-cycle arrest in early S phase. Exp Cell Res.

[271] Reitzer LJ, Magasanik B (1982). Asparagine synthetases of Klebsiella aerogenes: Properties and regulation of synthesis. J Bacteriol.

[272] Srikhanta YN, Atack JM, Beacham IR, Jennings MP (2013). Distinct physiological roles for the two L-asparaginase isozymes of Escherichia coli. Biochem Biophys Res Commun.

[273] Brigotti M, Rambelli F, Nanetti A, Zamboni M, Sperti S, Montanaro L (1990). Isolation of an inhibitor of cell-free protein synthesis from Salmonella enteritidis. Microbiologica.

[274] Bartalena L, Martino E, Antonelli A, Pacchiarotti A, Robbins J, Pinchera A (1986). Effect of the antileukemic agent L-asparaginase on thyroxine-binding globulin and albumin synthesis in cultured human hepatoma (HEP G2) cells. Endocrinology.

[275] Stahl PD, Wainszelbaum MJ (2009). Human-specific genes may offer a unique window into human cell signaling. Sci Signal.

[276] Kong C, Lange JJ, Samovski D, Su X, Liu J, Sundaresan S, Stahl PD (2013). Ubiquitination and degradation of the hominoid-specific oncoprotein TBC1D3 is regulated by protein palmitoylation. Biochem Biophys Res Commun.

[277] Frasa MA, Koessmeier KT, Ahmadian MR, Braga VM (2012). Illuminating the functional and structural repertoire of human TBC/RABGAPs. Nat Rev Mol Cell Biol.

[278] Pei L, Peng Y, Yang Y, Ling XB, Van Eyndhoven WG, Nguyen KC, Rubin M, Hoey T, Powers S, Li J (2002). PRC17, a novel oncogene encoding a Rab GTPase-activating protein, is amplified in prostate cancer. Cancer Res.

[279] Seaman MN, Harbour ME, Tattersall D, Read E, Bright N (2009). Membrane recruitment of the cargo-selective retromer subcomplex is catalysed by the small GTPase Rab7 and inhibited by the Rab-GAP TBC1D5. J Cell Sci.

[280] Popovic D, Dikic I (2014). TBC1D5 and the AP2 complex regulate ATG9 trafficking and initiation of autophagy. EMBO Rep.

[281] Frittoli E, Palamidessi A, Pizzigoni A, Lanzetti L, Garrè M, Troglio F, Troilo A, Fukuda M, Di Fiore PP, Scita G (2008). The primate-specific protein TBC1D3 is required for optimal macropinocytosis in a novel ARF6-dependent pathway. Mol Biol Cell.

[282] He Z, Tian T, Guo D, Wu H, Chen Y, Zhang Y, Wan Q, Zhao H, Wang C, Shen H (2014). Cytoplasmic retention of a nucleocytoplasmic protein TBC1D3 by microtubule network is required for enhanced EGFR signaling. PLoS One.

[283] Scheufele F, Wolf B, Kruse M, Hartmann T, Lempart J, Muehlich S, Pfeiffer AF, Field LJ, Charron MJ, Pan ZQ (2014). Evidence for a regulatory role of Cullin-RING E3 ubiquitin ligase 7 in insulin signaling. Cell Signal.

[284] Wainszelbaum MJ, Liu J, Kong C, Srikanth P, Samovski D, Su X, Stahl PD (2012). TBC1D3, a hominoid-specific gene, delays IRS-1 degradation and promotes insulin signaling by modulating p70 S6 kinase activity. PLoS One.

[285] Copps KD, White MF (2012). Regulation of insulin sensitivity by serine/threonine phosphorylation of insulin receptor substrate proteins IRS1 and IRS2. Diabetologia.

[286] Chantranupong L, Wolfson RL, Sabatini DM (2015). Nutrient-sensing mechanisms across evolution. Cell.

[287] Mirkin SM (2007). Expandable DNA repeats and human disease. Nature.

[288] Shaw G, Kamen R (1986). A conserved AU sequence from the 3′ untranslated region of GM-CSF mRNA mediates selective mRNA degradation. Cell.

[289] Uversky VN (2015). Functional roles of transiently and intrinsically disordered regions within proteins. FEBS J.

[290] Ragusa MJ, Dancheck B, Critton DA, Nairn AC, Page R, Peti W (2010). Spinophilin directs protein phosphatase 1 specificity by blocking substrate binding sites. Nat Struct Mol Biol.

[291] Nakanishi H, Obaishi H, Satoh A, Wada M, Mandai K, Satoh K, Nishioka H, Matsuura Y, Mizoguchi A, Takai Y (1997). Neurabin: A novel neural tissue-specific actin filament-binding protein involved in neurite formation. J Cell Biol.

[292] Chen Y, Liu Y, Cottingham C, McMahon L, Jiao K, Greengard P, Wang Q (2012). Neurabin scaffolding of adenosine receptor and RGS4 regulates anti-seizure effect of endogenous adenosine. J Neurosci.

[293] Kim SS, Wang H, Li XY, Chen T, Mercaldo V, Descalzi G, Wu LJ, Zhuo M (2011). Neurabin in the anterior cingulate cortex regulates anxiety-like behavior in adult mice. Mol Brain.

[294] Hu XD, Huang Q, Roadcap DW, Shenolikar SS, Xia H (2006). Actin-associated neurabin-protein phosphatase-1 complex regulates hippocampal plasticity. J Neurochem.

[295] Hu XD, Huang Q, Yang X, Xia H (2007). Differential regulation of AMPA receptor trafficking by neurabin-targeted synaptic protein phosphatase-1 in synaptic transmission and long-term depression in hippocampus. J Neurosci.

[296] Allen PB, Zachariou V, Svenningsson P, Lepore AC, Centonze D, Costa C, Rossi S, Bender G, Chen G, Feng J (2006). Distinct roles for spinophilin and neurabin in dopamine-mediated plasticity. Neuroscience.

[297] Wu LJ, Ren M, Wang H, Kim SS, Cao X, Zhuo M (2008). Neurabin contributes to hippocampal long-term potentiation and contextual fear memory. PLoS One.

[298] Finalet Ferreiro J, Rouhigharabaei L, Urbankova H, van der Krogt JA, Michaux L, Shetty S, Krenacs L, Tousseyn T, De Paepe P, Uyttebroeck A (2014). Integrative genomic and transcriptomic analysis identified candidate genes implicated in the pathogenesis of hepatosplenic T-cell lymphoma. PLoS One.

[299] Rowell JP, Simpson KL, Stott K, Watson M, Thomas JO (2012). HMGB1-facilitated p53 DNA binding occurs via HMG-Box/p53 transactivation domain interaction, regulated by the acidic tail. Structure.

[300] Teufel DP, Freund SM, Bycroft M, Fersht AR (2007). Four domains of p300 each bind tightly to a sequence spanning both transactivation subdomains of p53. Proc Natl Acad Sci USA.

[301] Zhang Z, Song M, Liu X, Kang SS, Kwon IS, Duong DM, Seyfried NT, Hu WT, Liu Z, Wang JZ (2014). Cleavage of tau by asparagine endopeptidase mediates the neurofibrillary pathology in Alzheimer's disease. Nat Med.

[302] Hsieh JJ, Cheng EH, Korsmeyer SJ (2003). Taspase1: A threonine aspartase required for cleavage of MLL and proper HOX gene expression. Cell.

[303] Aleksandrov AA, Kota P, Aleksandrov LA, He L, Jensen T, Cui L, Gentzsch M, Dokholyan NV, Riordan JR (2010). Regulatory insertion removal restores maturation, stability and function of DeltaF508 CFTR. J Mol Biol.

[304] Lewis HA, Zhao X, Wang C, Sauder JM, Rooney I, Noland BW, Lorimer D, Kearins MC, Conners K, Condon B (2005). Impact of the deltaF508 mutation in first nucleotide-binding domain of human cystic fibrosis transmembrane conductance regulator on domain folding and structure. J Biol Chem.

[305] Muchmore SW, Sattler M, Liang H, Meadows RP, Harlan JE, Yoon HS, Nettesheim D, Chang BS, Thompson CB, Wong SL (1996). X-ray and NMR structure of human Bcl-xL, an inhibitor of programmed cell death. Nature.

[306] Dho SH, Deverman BE, Lapid C, Manson SR, Gan L, Riehm JJ, Aurora R, Kwon KS, Weintraub SJ (2013). Control of cellular Bcl-xL levels by deamidation-regulated degradation. PLoS Biol.

[307] Lee JC, Kang SU, Jeon Y, Park JW, You JS, Ha SW, Bae N, Lubec G, Kwon SH, Lee JS (2012). Protein L-isoaspartyl methyltransferase regulates p53 activity. Nat Commun.

[308] Dawson R, Müller L, Dehner A, Klein C, Kessler H, Buchner J (2003). The N-terminal domain of p53 is natively unfolded. J Mol Biol.

[309] Schon O, Friedler A, Freund S, Fersht AR (2004). Binding of p53-derived ligands to MDM2 induces a variety of long range conformational changes. J Mol Biol.

[310] Rogers CS, Abraham WM, Brogden KA, Engelhardt JF, Fisher JT, McCray PB, McLennan G, Meyerholz DK, Namati E, Ostedgaard LS (2008). The porcine lung as a potential model for cystic fibrosis. Am J Physiol Lung Cell Mol Physiol.

[311] Seok J, Warren HS, Cuenca AG, Mindrinos MN, Baker HV, Xu W, Richards DR, McDonald-Smith GP, Gao H, Hennessy L (2013). Inflammation and Host Response to Injury, Large Scale Collaborative Research Program: Genomic responses in mouse models poorly mimic human inflammatory diseases. Proc Natl Acad Sci USA.

[312] Patterson PH (2009). Immune involvement in schizophrenia and autism: etiology, pathology and animal models. Behav Brain Res.

[313] Ohi N, Tokunaga A, Tsunoda H, Nakano K, Haraguchi K, Oda K, Motoyama N, Nakajima T (1999). A novel adenovirus E1B19K-binding protein B5 inhibits apoptosis induced by Nip3 by forming a heterodimer through the C-terminal hydrophobic region. Cell Death Differ.

[314] Zhang J, Loyd MR, Randall MS, Waddell MB, Kriwacki RW, Ney PA (2012). A short linear motif in BNIP3L (NIX) mediates mitochondrial clearance in reticulocytes. Autophagy.

[315] Perutz M (1994). Polar zippers: their role in human disease. Protein Sci.

[316] Perutz MF, Pope BJ, Owen D, Wanker EE, Scherzinger E (2002). Aggregation of proteins with expanded glutamine and alanine repeats of the glutamine-rich and asparagine-rich domains of Sup35 and of the amyloid beta-peptide of amyloid plaques. Proc Natl Acad Sci USA.

[317] Simon M, Hancock JM (2009). Tandem and cryptic amino acid repeats accumulate in disordered regions of proteins. Genome Biol.

[318] Tompa P (2003). Intrinsically unstructured proteins evolve by repeat expansion. Bioessays.

[319] Li L, Moore PK (2007). An overview of the biological significance of endogenous gases: New roles for old molecules. Biochem Soc Trans.

[320] Levine SM, Rosen A, Casciola-Rosen LA (2003). Anti-aminoacyl tRNA synthetase immune responses: Insights into the pathogenesis of the idiopathic inflammatory myopathies. Curr Opin Rheumatol.

[321] Beaulande M, Tarbouriech N, Härtlein M (1998). Human cytosolic asparaginyl-tRNA synthetase: cDNA sequence, functional expression in Escherichia coli and characterization as human autoantigen. Nucleic Acids Res.

[322] Howard OM, Dong HF, Yang D, Raben N, Nagaraju K, Rosen A, Casciola-Rosen L, Härtlein M, Kron M, Yang D (2002). Histidyl-tRNA synthetase and asparaginyl-tRNA synthetase, autoantigens in myositis, activate chemokine receptors on T lymphocytes and immature dendritic cells. J Exp Med.

[323] Park SJ, Kim SH, Choi HS, Rhee Y, Lim SK (2009). Fibroblast growth factor 2-induced cytoplasmic asparaginyl-tRNA synthetase promotes survival of osteoblasts by regulating anti-apoptotic PI3K/Akt signaling. Bone.

[324] Kron MA, Wang C, Vodanovic-Jankovic S, Howard OM, Kuhn LA (2012). Interleukin-8-like activity in a filarial asparaginyl-tRNA synthetase. Mol Biochem Parasitol.

[325] Bonfils G, Jaquenoud M, Bontron S, Ostrowicz C, Ungermann C, De Virgilio C (2012). Leucyl-tRNA synthetase controls TORC1 via the EGO complex. Mol Cell.

[326] Avruch J, Long X, Ortiz-Vega S, Rapley J, Papageorgiou A, Dai N (2009). Amino acid regulation of TOR complex 1. Am J Physiol Endocrinol Metab.

[327] Hara K, Yonezawa K, Weng QP, Kozlowski MT, Belham C, Avruch J (1998). Amino acid sufficiency and mTOR regulate p70 S6 kinase and eIF-4E BP1 through a common effector mechanism. J Biol Chem.

[328] Wang S, Tsun ZY, Wolfson RL, Shen K, Wyant GA, Plovanich ME, Yuan ED, Jones TD, Chantranupong L, Comb W (2015). Metabolism. Lysosomal amino acid transporter SLC38A9 signals arginine sufficiency to mTORC1. Science.

[329] Rebsamen M, Pochini L, Stasyk T, de Araújo ME, Galluccio M, Kandasamy RK, Snijder B, Fauster A, Rudashevskaya EL, Bruckner M (2015). SLC38A9 is a component of the lysosomal amino acid sensing machinery that controls mTORC1. Nature.

[330] Bar-Peled L, Sabatini DM (2014). Regulation of mTORC1 by amino acids. Trends Cell Biol.

[331] Efeyan A, Zoncu R, Sabatini DM (2012). Amino acids and mTORC1: From lysosomes to disease. Trends Mol Med.

[332] Abraham RT (2015). Cell biology. Making sense of amino acid sensing. Science.

[333] Weng L, Quinlivan E, Gong Y, Beitelshees AL, Shahin MH, Turner ST, Chapman AB, Gums JG, Johnson JA, Frye RF (2015). Association of branched and aromatic amino acids levels with metabolic syndrome and impaired fasting glucose in hypertensive patients. Metab Syndr Relat Disord.

[334] Björkegren JL, Kovacic JC, Dudley JT, Schadt EE (2015). Genome-wide significant loci: How important are they? Systems genetics to understand heritability of coronary artery disease and other common complex disorders. J Am Coll Cardiol.

[335] Zhang X, Bailey SD, Lupien M (2014). Laying a solid foundation for Manhattan - 'setting the functional basis for the post-GWAS era'. Trends Genet.

[336] Gusev A, Bhatia G, Zaitlen N, Vilhjalmsson BJ, Diogo D, Stahl EA, Gregersen PK, Worthington J, Klareskog L, Raychaudhuri S (2013). Quantifying missing heritability at known GWAS loci. PLoS Genet.

[337] Manolio TA, Collins FS, Cox NJ, Goldstein DB, Hindorff LA, Hunter DJ, McCarthy MI, Ramos EM, Cardon LR, Chakravarti A (2009). Finding the missing heritability of complex diseases. Nature.

[338] Cross-Disorder Group of the Psychiatric Genomics Consortium (2013). Identification of risk loci with shared effects on five major psychiatric disorders: A genome-wide analysis. Lancet.

[339] Nikoletopoulou V, Lickert H, Frade JM, Rencurel C, Giallonardo P, Zhang L, Bibel M, Barde YA (2010). Neurotrophin receptors TrkA and TrkC cause neuronal death whereas TrkB does not. Nature.

[340] Yoon K, Jang HD, Lee SY (2004). Direct interaction of Smac with NADE promotes TRAIL-induced apoptosis. Biochem Biophys Res Commun.

[341] Zhang CK, Stein PB, Liu J, Wang Z, Yang R, Cho JH, Gregersen PK, Aerts JM, Zhao H, Pastores GM (2012). Genome-wide association study of N370S homozygous Gaucher disease reveals the candidacy of CLN8 gene as a genetic modifier contributing to extreme phenotypic variation. Am J Hematol.

[342] Bultron G, Kacena K, Pearson D, Boxer M, Yang R, Sathe S, Pastores G, Mistry PK (2010). The risk of Parkinson's disease in type 1 Gaucher disease. J Inherit Metab Dis.

[343] Urano M, Nagao T, Miyabe S, Ishibashi K, Higuchi K, Kuroda M (2015). Characterization of mammary analogue secretory carcinoma of the salivary gland: Discrimination from its mimics by the presence of the ETV6-NTRK3 translocation and novel surrogate markers. Hum Pathol.

[344] Lannon CL, Sorensen PH (2005). ETV6-NTRK3: A chimeric protein tyrosine kinase with transformation activity in multiple cell lineages. Semin Cancer Biol.

[345] Genevois AL, Ichim G, Coissieux MM, Lambert MP, Lavial F, Goldschneider D, Jarrosson-Wuilleme L, Lepinasse F, Gouysse G, Herceg Z (2013). Dependence receptor TrkC is a putative colon cancer tumor suppressor. Proc Natl Acad Sci USA.

[346] Luo Y, Kaz AM, Kanngurn S, Welsch P, Morris SM, Wang J, Lutterbaugh JD, Markowitz SD, Grady WM (2013). NTRK3 is a potential tumor suppressor gene commonly inactivated by epigenetic mechanisms in colorectal cancer. PLoS Genet.

[347] Ivanov SV, Panaccione A, Brown B, Guo Y, Moskaluk CA, Wick MJ, Brown JL, Ivanova AV, Issaeva N, El-Naggar AK (2013). TrkC signaling is activated in adenoid cystic carcinoma and requires NT-3 to stimulate invasive behavior. Oncogene.

[348] Kim MS, Kim GM, Choi YJ, Kim HJ, Kim YJ, Jin W (2013). TrkC promotes survival and growth of leukemia cells through Akt-mTOR-dependent up-regulation of PLK-1 and Twist-1. Mol Cells.

[349] Weinkauf C, Salvador R, Pereiraperrin M (2011). Neurotrophin receptor TrkC is an entry receptor for Trypanosoma cruzi in neural, glial, and epithelial cells. Infect Immun.

[350] Capewell P, Cooper A, Clucas C, Weir W, Macleod A (2015). A co-evolutionary arms race: Trypanosomes shaping the human genome, humans shaping the trypanosome genome. Parasitology.

[351] Marx SO, Reiken S, Hisamatsu Y, Jayaraman T, Burkhoff D, Rosemblit N, Marks AR (2000). PKA phosphorylation dissociates FKBP12.6 from the calcium release channel (ryanodine receptor): Defective regulation in failing hearts. Cell.

[352] Zhou L, He M, Mo Z, Wu C, Yang H, Yu D, Yang X, Zhang X, Wang Y, Sun J (2013). A genome wide association study identifies common variants associated with lipid levels in the Chinese population. PLoS One.

[353] Del-Aguila JL, Beitelshees AL, Cooper-Dehoff RM, Chapman AB, Gums JG, Bailey K, Gong Y, Turner ST, Johnson JA, Boerwinkle E (2014). Genome-wide association analyses suggest NELL1 influences adverse metabolic response to HCTZ in African Americans. Pharmacogenomics J.

[354] Jeong SW, Chung M, Park SJ, Cho SB, Hong KW (2014). Genome-wide association study of metabolic syndrome in koreans. Genomics Inform.

[355] Wellcome Trust Case Control Consortium (2007). Genome-wide association study of 14,000 cases of seven common diseases and 3,000 shared controls. Nature.

[356] Eirís N, González-Lara L, Santos-Juanes J, Queiro R, Coto E, Coto-Segura P (2014). Genetic variation at IL12B, IL23R and IL23A is associated with psoriasis severity, psoriatic arthritis and type 2 diabetes mellitus. J Dermatol Sci.

[357] Zhang M, Cai ZR, Zhang B, Cai X, Li W, Guo Z, Ma L (2014). Functional polymorphisms in interleukin-23 receptor and susceptibility to coronary artery disease. DNA Cell Biol.

[358] Mizuki N, Meguro A, Ota M, Ohno S, Shiota T, Kawagoe T, Ito N, Kera J, Okada E, Yatsu K (2010). Genome-wide association studies identify IL23R-IL12RB2 and IL10 as Behçet's disease susceptibility loci. Nat Genet.

[359] Daryabor G, Mahmoudi M, Jamshidi A, Nourijelyani K, Amirzargar A, Ahmadzadeh N, Farhadi E, Nicknam MH (2014). Determination of IL-23 receptor gene polymorphism in Iranian patients with ankylosing spondylitis. Eur Cytokine Netw.

[360] Zhang F, Liu H, Chen S, Low H, Sun L, Cui Y, Chu T, Li Y, Fu X, Yu Y (2011). Identification of two new loci at IL23R and RAB32 that influence susceptibility to leprosy. Nat Genet.

[361] Hornakova T, Staerk J, Royer Y, Flex E, Tartaglia M, Constantinescu SN, Knoops L, Renauld JC (2009). Acute lymphoblastic leukemia-associated JAK1 mutants activate the Janus kinase/STAT pathway via interleukin-9 receptor alpha homodimers. J Biol Chem.

[362] Leonard WJ (1994). The defective gene in X-linked severe combined immunodeficiency encodes a shared interleukin receptor subunit: Implications for cytokine pleiotropy and redundancy. Curr Opin Immunol.

[363] Baba A, Ohtake F, Okuno Y, Yokota K, Okada M, Imai Y, Ni M, Meyer CA, Igarashi K, Kanno J (2011). PKA-dependent regulation of the histone lysine demethylase complex PHF2-ARID5B. Nat Cell Biol.

[364] Papaemmanuil E, Hosking FJ, Vijayakrishnan J, Price A, Olver B, Sheridan E, Kinsey SE, Lightfoot T, Roman E, Irving JA (2009). Loci on 7p12.2, 10q21.2 and 14q11.2 are associated with risk of childhood acute lymphoblastic leukemia. Nat Genet.

[365] Chokkalingam AP, Hsu LI, Metayer C, Hansen HM, Month SR, Barcellos LF, Wiemels JL, Buffler PA (2013). Genetic variants in ARID5B and CEBPE are childhood ALL susceptibility loci in Hispanics. Cancer Causes Control.

[366] Xu H, Cheng C, Devidas M, Pei D, Fan Y, Yang W, Neale G, Scheet P, Burchard EG, Torgerson DG (2012). ARID5B genetic polymorphisms contribute to racial disparities in the incidence and treatment outcome of childhood acute lymphoblastic leukemia. J Clin Oncol.

[367] Gutiérrez-Camino Á, López-López E, Martín-Guerrero I, Sánchez-Toledo J, García de Ann N, Carboné Bañeres A, García-Miguel P, Navajas A, García-Orad Á (2013). Intron 3 of the ARID5B gene: A hot spot for acute lymphoblastic leukemia susceptibility. J Cancer Res Clin Oncol.

[368] Guo LM, Xi JS, Ma Y, Shao L, Nie CL, Wang GJ (2014). ARID5B gene rs10821936 polymorphism is associated with childhood acute lymphoblastic leukemia: a meta-analysis based on 39,116 subjects. Tumour Biol.

[369] Lin CY, Li MJ, Chang JG, Liu SC, Weng T, Wu KH, Yang SF, Huang FK, Lo WY, Peng CT (2014). High-resolution melting analyses for genetic variants in ARID5B and IKZF1 with childhood acute lymphoblastic leukemia susceptibility loci in Taiwan. Blood Cells Mol Dis.

[370] Lu Y, Vitart V, Burdon KP, Khor CC, Bykhovskaya Y, Mirshahi A, Hewitt AW, Koehn D, Hysi PG, Ramdas WD (2013). NEIGHBOR Consortium: Genome-wide association analyses identify multiple loci associated with central corneal thickness and keratoconus. Nat Genet.

[371] Engel SM, Joubert BR, Wu MC, Olshan AF, Håberg SE, Ueland PM, Nystad W, Nilsen RM, Vollset SE, Peddada SD (2014). Neonatal genome-wide methylation patterns in relation to birth weight in the Norwegian Mother and Child Cohort. Am J Epidemiol.

[372] Newton-Cheh C, Johnson T, Gateva V, Tobin MD, Bochud M, Coin L, Najjar SS, Zhao JH, Heath SC, Eyheramendy S (2009). Wellcome Trust Case Control Consortium: Genome-wide association study identifies eight loci associated with blood pressure. Nat Genet.

[373] Okada Y, Terao C, Ikari K, Kochi Y, Ohmura K, Suzuki A, Kawaguchi T, Stahl EA, Kurreeman FA, Nishida N (2012). Meta-analysis identifies nine new loci associated with rheumatoid arthritis in the Japanese population. Nat Genet.

[374] Drago A, Giegling I, Schäfer M, Hartmann AM, Konte B, Friedl M, Serretti A, Rujescu D (2014). Genome-wide association study supports the role of the immunological system and of the neurodevelopmental processes in response to haloperidol treatment. Pharmacogenet Genomics.

[375] Yang W, Tang H, Zhang Y, Tang X, Zhang J, Sun L, Yang J, Cui Y, Zhang L, Hirankarn N (2013). Meta-analysis followed by replication identifies loci in or near CDKN1B, TET3, CD80, DRAM1, and ARID5B as associated with systemic lupus erythematosus in Asians. Am J Hum Genet.

[376] Whitson RH, Tsark W, Huang TH, Itakura K (2003). Neonatal mortality and leanness in mice lacking the ARID transcription factor Mrf-2. Biochem Biophys Res Commun.

[377] Yamakawa T, Sugimoto K, Whitson RH, Itakura K (2010). Modulator recognition factor-2 regulates triglyceride metabolism in adipocytes. Biochem Biophys Res Commun.

[378] Wang G, Watanabe M, Imai Y, Hara K, Manabe I, Maemura K, Horikoshi M, Ozeki A, Itoh C, Sugiyama T (2012). Associations of variations in the MRF2/ARID5B gene with susceptibility to type 2 diabetes in the Japanese population. J Hum Genet.

[379] Urayama KY, Chokkalingam AP, Manabe A, Mizutani S (2013). Current evidence for an inherited genetic basis of childhood acute lymphoblastic leukemia. Int J Hematol.

[380] Prakash T, Sharma VK, Adati N, Ozawa R, Kumar N, Nishida Y, Fujikake T, Takeda T, Taylor TD (2010). Expression of conjoined genes: Another mechanism for gene regulation in eukaryotes. PLoS One.

[381] Geer LY, Marchler-Bauer A, Geer RC, Han L, He J, He S, Liu C, Shi W, Bryant SH (2010). The NCBI BioSystems database. Nucleic Acids Res.

[382] Parge HE, Arvai AS, Murtari DJ, Reed SI, Tainer JA (1993). Human CksHs2 atomic structure: A role for its hexameric assembly in cell cycle control. Science.

[383] Liberal V, Martinsson-Ahlzén HS, Liberal J, Spruck CH, Widschwendter M, McGowan CH, Reed SI (2012). Cyclin-dependent kinase subunit (Cks) 1 or Cks2 overexpression overrides the DNA damage response barrier triggered by activated oncoproteins. Proc Natl Acad Sci USA.

[384] Agirre X, Román-Gómez J, Jiménez-Velasco A, Garate L, Montiel-Duarte C, Navarro G, Vázquez I, Zalacain M, Calasanz MJ, Heiniger A (2006). ASPP1, a common activator of TP53, is inactivated by aberrant methylation of its promoter in acute lymphoblastic leukemia. Oncogene.

[385] Khattar V, Thottassery JV (2013). Cks1: Structure, emerging roles and implications in multiple cancers. J Cancer Ther.

[386] Lee SW, Lin CY, Tian YF, Sun DP, Lin LC, Chen LT, Hsing CH, Huang CT, Hsu HP, Huang HY (2014). Overexpression of CDC28 protein kinase regulatory subunit 1B confers an independent prognostic factor in nasopharyngeal carcinoma. APMIS.

[387] Vigneron AM, Vousden KH (2012). An indirect role for ASPP1 in limiting p53-dependent p21 expression and cellular senescence. EMBO J.

[388] Valaperta R, Rizzo V, Lombardi F, Verdelli C, Piccoli M, Ghiroldi A, Creo P, Colombo A, Valisi M, Margiotta E (2014). Adenine phosphoribosyltransferase (APRT) deficiency: Identification of a novel nonsense mutation. BMC Nephrol.

[389] Ibrahim L, Aladle D, Mansour A, Hammad A, Al Wakeel AA, Abd El-Hameed SA (2014). Expression and prognostic significance of livin/BIRC7 in childhood acute lymphoblastic leukemia. Med Oncol.

[390] Mulcahy ME, Geoghegan JA, Monk IR, O'Keeffe KM, Walsh EJ, Foster TJ, McLoughlin RM (2012). Nasal colonisation by Staphylococcus aureus depends upon clumping factor B binding to the squamous epithelial cell envelope protein loricrin. PLoS Pathog.

[391] Hawkes WC, Wang TT, Alkan Z, Richter BD, Dawson K (2009). Selenoprotein W modulates control of cell cycle entry. Biol Trace Elem Res.

[392] Pekarsky Y, Drusco A, Kumchala P, Croce CM, Zanesi N (2015). The long journey of TCL1 transgenic mice: Lessons learned in the last 15 years. Gene Expr.

[393] Chalouhi N, Theofanis T, Starke RM, Zanaty M, Jabbour P, Dooley SA, Hasan D (2015). Potential role of granulocyte-monocyte colony-stimulating factor in the progression of intracranial aneurysms. DNA Cell Biol.

[394] Small KS, Hedman AK, Grundberg E, Nica AC, Thorleifsson G, Kong A, Thorsteindottir U, Shin SY, Richards HB, Soranzo N (2011). GIANT Consortium; MAGIC Investigators; DIAGRAM Consortium; MuTHER Consortium: Identification of an imprinted master trans regulator at the KLF14 locus related to multiple metabolic phenotypes. Nat Genet.

